# Liquid *vs* Solid Culture Medium to Evaluate Proportion and Time to Change in Management of Suspects of Tuberculosis—A Pragmatic Randomized Trial in Secondary and Tertiary Health Care Units in Brazil

**DOI:** 10.1371/journal.pone.0127588

**Published:** 2015-06-05

**Authors:** Adriana da Silva Rezende Moreira, Gisele Huf, Maria Armanda Monteiro da Silva Vieira, Paulo Albuquerque da Costa, Fábio Aguiar, Anna Grazia Marsico, Leila de Souza Fonseca, Mônica Ricks, Martha Maria Oliveira, Anne Detjen, Paula Isono Fujiwara, Stephen Bertel Squire, Afranio Lineu Kritski

**Affiliations:** 1 Tuberculosis Academic Program, Medical School, Federal University of Rio de Janeiro, Rio de Janeiro, Brazil; 2 Policlínica Augusto Amaral Peixoto, Guadalupe/SMS, Rio de Janeiro, Brazil; 3 International Union Against Tuberculosis and Lung Disease, Paris, France; 4 Liverpool School of Tropical Medicine, Liverpool, United Kingdom; Glaxo Smith Kline, DENMARK

## Abstract

**Background:**

The use of liquid medium (MGIT960) for tuberculosis (TB) diagnosis was recommended by WHO in 2007. However, there has been no evaluation of its effectiveness on clinically important outcomes.

**Methods and Findings:**

A pragmatic trial was carried out in a tertiary hospital and a secondary health care unit in Rio de Janeiro City, Brazil. Participants were 16 years or older, suspected of having TB. They were excluded if only cerebral spinal fluid or blood specimens were available for analysis. MGIT960 technique was compared with the Lowenstein-Jensen (LJ) method for laboratory diagnosis of active TB. Primary outcome was the proportion of patients who had their initial medical management changed within 2 months after randomisation. Secondary outcomes were: mean time for changing the procedure, patient satisfaction with the overall treatment and adverse events. Data were analysed by intention-to-treat. Between April 2008 and September 2011, 693 patients were enrolled (348 to MGIT, 345 to LJ). Smear and culture results were positive for 10% and 15.7% of participants, respectively. Patients in the MGIT arm had their initial medical management changed more frequently than those in the LJ group (10.1% MGIT vs 3.8% LJ, RR 2.67 95% CI 1.44–.96, p = 0.002, NNT 16, 95% CI 10–39). Mean time for changing the initial procedure was greater in LJ group at both sites: 20.0 and 29.6 days in MGIT group and 52.2 and 64.3 in LJ group (MD 33.5, 95% CI 30.6–36.4, p = 0.0001). No other important differences were observed.

**Conclusions:**

This study suggests that opting for the MGIT960 system for TB diagnosis provides a promising case management model for improving the quality of care and control of TB.

**Trial Registration:**

Controlled-Trials.com ISRCTN79888843

## Introduction

Tuberculosis (TB) is one of the world’s leading infectious diseases. In 2011, nearly 9 million people fell ill from TB, and 1.4 million people died [1]. In 2006, the World Health Organization’s Global Plan to Stop TB prioritized improving diagnosis and treatment to improve control of the disease [2]. In 2007, approximately 20–30% of the patients treated in low-income countries were treated for TB without bacteriological confirmation.

Despite the fact that acid fast bacilli sputum smear microscopy has a low sensitivity (60%), it remains the most frequently used test for the diagnosis of pulmonary TB in low-income countries [3,4]. The acid fast bacilli smear sensitivity is even lower in (human immunodeficiency virus (HIV)—infected or immunosuppressed patients and in children (<30%) [3].

In most high-burden countries, mycobacterial culture is performed on solid Lowenstein-Jensen (LJ) medium. The LJ culture method has a higher sensitivity than the acid fast bacilli smear (80–85%), but due to the long incubation time (4–6 weeks), several additional weeks are required for results to be available [4]. In order to respond more effectively to the emergence of TB and HIV co-infections and multi-drug resistant TB, in 2007 the World Health Organization recommended that new TB diagnostic technologies, such as liquid culture, be used for the detection of *Mycobacterium tuberculosis (M*.*tb)*. This recommendation was based on a review of the available scientific evidence and expert consultation [5–7]. More recently, liquid medium has become a standard reference for TB diagnosis [3].

New recommendations published by the World Health Organization are rapidly incorporated into clinical guidelines in middle-income countries [8]. These recommendations are usually incorporated with few changes or adjustments to the local healthcare needs of each country [8]. Each diagnostic strategy affect subsequent clinical decisions and authors advocate for clinical trials to evaluate the advantages and disadvantages that these strategies have on the decision-making process [9–12].

To assist with the incorporation of liquid culture for *M*.*tb* detection into the Unified Health System in Brazil [13,14], the International Union against Tuberculosis and Lung Disease (The Union), the Brazilian Network of Tuberculosis Research (Rede TB) and the Academic Tuberculosis Program of the Federal University of Rio de Janeiro carried out a pragmatic clinical trial.

## Methods

### Setting

We conducted a multicenter, open-label, two-arm trial with inpatients from the University Hospital Clementino Fraga Filho (HUCFF) and outpatients from Policlínica Augusto Amaral Peixoto (PAAP) in Rio de Janeiro, Brazil (TB incidence 100/100,000). HUCFF is a tertiary teaching hospital of the Federal University of Rio de Janeiro. It is an HIV Reference Center with 494 beds distributed among 26 disciplines (46 admissions per day). PAAP is a secondary level health unit with a high TB incidence located in the northern zone of the city of Rio de Janeiro. This facility serves 300,000 inhabitants and treats 200 TB patients per year (6% infected with HIV).

### Participants

Eligible participants were any patients 16 years or older for whom an examination for TB diagnosis was requested. Individuals were excluded if they were already receiving anti-TB treatment, if they had only cerebral spinal fluid or blood specimens for analysis, or if they refused to give a signed informed written consent. For minors, informed written consent was obtained from the guardians on behalf of the participant. At PAAP, the study began in April 2008 and ended in February 2010; at HUCFF, recruitment started in April 2008 and finished in September 2011. Follow up ended six months after these dates.

### Interventions

Participants were randomly assigned to have their samples analysed by the BACTEC MGIT 960 Mycobacterial Detection System (MGIT960—Mycobacteria Growth Indicator Tube) or by the LJ proportion method according to the Brazilian Tuberculosis National Guidelines [14]. All clinical samples from both sites were sent to the University Hospital Mycobacteriology Laboratory for culture, drug susceptibility testing and identification at species level. Tests were performed according to the hospital’s laboratory routine, and the techniques are fully described elsewhere [15,16]. The smears were stained by Ziehl-Neelsen at the University Hospital Mycobacteriology Laboratory and scored according to international guidelines.

TB cases were defined as those with a positive M.tb culture from a clinical specimen (bacteriological confirmation) or those who had had clinical and radiological improvement after six months of anti-TB treatment without the treatment of other diseases. This clinical and radiological improvement was blindly reviewed by three different chest physicians who were not involved in the study in any other way. Non-TB was considered in patients whose acid fast smear and culture for M.tb were negative, and with no clinical and radiological evolution compatible with active TB.

### Randomisation and masking procedures

The two sites were randomised separately at a 1:1 ratio in blocks of 4 and 6 in random order within which random sequences of treatments were generated using the ‘List Randomizer’ option from http://www.random.org/. The study was open, but allocation was fully concealed and undertaken by personnel not involved in the clinical interface. Consecutively numbered, sealed, fully opaque envelopes that were identical in every way to the outside observer were prepared. Each contained information regarding which test should be conducted. These envelopes were held in locked drawers and were opened by the trial researchers. The disease probabilities of TB and drug-resistant TB were recorded for each person before the envelope was opened and the test allocation was known. Using a standard form, a trained nurse with experience in diagnosing TB classified eligible individuals into the following risk categories: low (≤25%); intermediate (26%-75%); and high (>75%). These categories estimated the disease probability based on clinical history and physical examination, (cough, fever, weight loss, etc…). This score was only used to check the success of randomisation. At PAAP, a history of tobacco smoking was ascertained at entry by a standardized, routinely performed, staff-administered questionnaire (current smoker/ past smoker/never smoker). Alcohol abuse was identified by the CAGE (**C**ut down, **A**nnoyed, **G**uilty, **E**ye-opener) criteria [17]. The patient care during the study did not deviate from routinely administered procedures, with the exception of the choice of the laboratory test.

### Outcomes

The primary outcome was defined as the proportion of patients in each group whose initial prescription changed by the second month after randomization. The ‘change’ could be initiating or stopping TB treatment. The secondary outcomes were the mean time to changing the initial clinical prescription, the proportion of patients with positive culture and positive smear microscopy for TB, the proportion of patients initiating treatment before test results, important adverse events and patient satisfaction with the overall treatment. This last variable was measured by asking patients the following question six months after randomisation or at the time of discharge: “how did you feel about your treatment: pleased, neutral, or not pleased?” For patients with positive bacteriological test results, we estimated the proportion of bacteriological conversion at 2, 4 and 6 months.

### Sample size

Concerning the primary outcome, the smallest absolute difference considered important by a panel of specialists working at HUCFF was 10%. Considering an 80% chance of detecting this difference at the 5% level of statistical significance, we intended to recruit at least 770 participants.

### Statistical analysis

We compared the socio-demographic and clinical characteristics between groups at trial entry. For dichotomous outcomes, we calculated the proportions for both groups, and for continuous outcomes, we calculated the means, standard deviations and medians. We used intention-to-treat analysis to calculate the relative risk, the risk difference, the number needed to treat and the 95% confidence intervals for these parameters. No subgroup analysis was pre-specified. All analyses were performed using SPSS software (version 17).

The protocol was approved by the Federal University Research Ethics Committee in May, 2007. All participants gave written informed consent. The study registry was obtained after participant recruitment began because the Brazilian Clinical Trial registry only became mandatory in 2012. The authors confirm that all ongoing and related trials for this intervention are registered.

### Deviations from protocol

We had hoped to record bacteriological conversion at 2, 4 and 6 months, but, it was only checked at 6 months at PAAP. Additionally, the participants’ satisfaction was not recorded at PAAP. Furthermore, recruitment was stopped early at both of the sites for different reasons. At PAAP, a new director did not support the continuation of the study, and at HUCFF, demolition of part of the building resulted in the suspension of medical care services from November 1^st^, 2010 to March 1^st^, 2011.

## Results

Overall, 893 people suspected of having TB were assessed for eligibility, and 693 were enrolled (348 to MGIT and 345 to LJ). S1 Fig presents the flow diagram for participants at both sites. At HUCFF, one patient was lost to the primary outcome (LJ group) because he was erroneously randomised after taking TB drugs for 30 days. Physicians in the hospital agreed that after 30 days of treatment, patient’s treatment should not be stopped even with negative results, so his inclusion in the study did not impact the primary outcome analysis. He was analysed as not having changed procedure. At PAAP, one patient was also lost to the primary outcome (MGIT group). His examination was not performed because the material he provided twice was not suitable for analysis. His smear microscopy was negative and he was not treated for TB.

Full characteristics of the patients at study entry are described in Table 1. The randomisation process was successful in having very similar groups with regard to sex, mean age, guessed probability of TB and MDR-TB (multidrug resistant) and the proportion of subjects infected with HIV. At HUCFF, data on smoking and drinking habits are not routinely collected, and at PAAP, HIV examination is not routinely performed.

**Table 1:** Patient characteristics at study entry.

	HUCFF		PAAP	
	N (%)		N (%)	
	MGIT	LJ	MGIT	LJ
	(N = 214)	(N = 213)	(N = 133)	(N = 132)
**Male**	123 (57.5)	123 (57.7)	72 (53.7)	74 (56.1)
Mean age years (SD)*	51.1 (15.6)	50.8 (17.9)	45.5 (16.6)	44.7 (14.3)
**Probable drug susceptible TB**				
High	44 (20.6)	41 (19.2)	5 (3.7)	6 (4.5)
Medium	110 (51.4)	107 (50.2)	81 (60.4)	82 (62.1)
Low	60 (28.0)	65 (30.5)	48 (35.8)	44 (33.3)
**Probable drug resistant TB**				
High	3 (1.4)	2 (0.9)	4 (3.0)	4 (3.0)
Medium	41 (19.2)	37 (17.4)	17 (12.7)	25 (18.9)
Low	170 (79.4)	174 (81.7)	113 (84.3)	103 (78.0)
**HIV+**				
Yes	69 (32.2)	65 (30.5)	-	-
No	100 (46.7)	102 (47.9)	-	-
unknown	45 (21.0)	46 (21.6)	-	-
**Never smoked**	-	-	47 (36.7)	46 (36.2)
**Alcoholism**	**-**	-	29 (22.7)	29 (22.8)

Overall, the smear results were positive for 10% of the participants (35/348 MGIT vs 34/345 LJ), and active pulmonary TB was bacteriologically diagnosed in 15.7% (58/348 MGIT vs 51/345 LJ). At PAAP, 2 clinical specimens were evaluated per patient in both arms. For the MGIT960 arm at HUCFF, 2, 3 and 4 clinical specimens were evaluated for 73, 31 and 4 patients, respectively, and for the LJ arm, those figures were 72, 37 and 7 patients, respectively. At HUCFF, 80 participants on LJ arm and 72 at MGIT arm had also extrapulmonary specimens tested for TB. At PAAP, three patients suspected of having also extrapulmonary infections were referred to proceed the testing elsewhere. The mean time for a final TB diagnosis was 37.7 days for MGIT (95% CI 36.2–39.1, SD 10.6, median 42 and range 4–42) and 55.1 days for LJ (95% CI 53.8–56.5, SD 9.9, median 59 and range 19–60). The mean time for positive results was 10.7 days for MGIT (95% CI 8.4–13.1, SD 5.7, median 9 and range 4–25) and 31.7 days for LJ (95% CI 27.6–35.7, SD 8.9, median 31 and range 19–49). Under field conditions, physicians at HUCFF had immediate access to TB test results while at PAAP test results are manually delivered twice a week. Just over 18% of the patients were treated for TB (65/348 MGIT vs 62/345 LJ), and 14 participants at HUCFF had solely extra-pulmonary TB (Table 2). Twelve percent of those suspected of having TB initiated treatment before the test results were available (34/348 MGIT vs 50/345 LJ, RR 0.67 95% CI 0.45–1.01, p = 0.06). Regarding the primary outcome, people allocated to MGIT had their initial treatment changed more frequently after test results compared to those allocated to LJ (35/348 MGIT vs 13/345 LJ, RR 2.67, 95% CI 1.44–4.96, p = 0.002, NNT 16, 95% CI 10–39). Although the majority began TB treatment, nine patients began non-mycobacterial tuberculosis (NMT) treatment, and two stopped TB treatment based on their physicians’ decisions.

**Table 2:** Clinical and laboratory outcomes according to study group and health unit.

	HUCFF		PAAP		Total	RR (95%IC)	p-value
	N (%)		N (%)		MGIT vs LJ		
	MGIT = 214	LJ = 213	MGIT = 134	LJ = 132			
**Smear microscopy**							
Positive	6 (2.8)	10 (4.7)	29 (21.6)	24 (19.0)	35 (10.1%) *vs* 34 (9.8%)	1.02 (0.65–1.60)	0.97
Not performed	4 (1.9)	2 (0.9)		0			
**Culture**							
MTB	25 (11.7)	20 (9.4)	33 (24.6)	31 (23.5)	58 (16.7%) *vs* 51 (14.8%)	1.13 (0.80–1.59)	0.56
NTM	2 (0.9)	2 (0.9)	5 (3.7)	2 (1.5)	7 (2.0%) *vs* 4 (1.2%)	1.73 (0.51–5.89)	0.38
Contamination	6 (2.8)	7 (3.3)	0	1 (0.7)			
Total (NTM & MTB)	27 (12.6)	22 (10.3)	38 (28.3)	33 (25.0)	65 (18.7%) *vs* 55 (15.9%)	1.17 (0.84–1.62)	0.34
**Treated for TB**	27 (12.6)	23 (10.8)	38 (29.0)	39 (30.0)	65 (18.7%) *vs* 62 (18.0%)	1.00 (0.73–1.38)	0.96
**Location of TB**							
Pulmonary	14 (6.5)	15 (7.0)	38 (28.8)	36 (27.2)	52 (14.9%) *vs* 51 (14.8%)	1.01 (0.71–1.44)	0.96
Extra-pulm	10 (4.7)	4 (1.9)	0	0	10 (2.9%) *vs* 4 (1.2%)	2.48 (0.79–7.83)	0.12
Pulm+extrapulm	4 (1.9)	3 (1.4)	0	3 (2.3)	4 (1.2%) *vs* 6 (1.7%)	0.66 (0.19–2.32)	0.52
**Initiated anti-TB treatment before test result**	13 (6.1)	17 (8.0)	21 (16.0)	33 (25.0)	34 (9.8%) *vs* 50 (14.5%)	0.67 (0.45–1.01)	0.06
**Changed procedure after test result**	17 (7.9)	7 (3.3)	18 (13.4)	6 (5.0)	35 (10.1%) *vs* 13 (3.8%)	2.67 (1.44–4.96)	0.002
**Which changing**							
Beginning treatment for TB	14 (6.5)	6 (2.8)	13 (9.7)	4 (3.0)			
Beginning treatment for NTM	2 (0.9)	1 (0.5)	4 (2.9)	2 (1.5)			
Stopping treatment	1 (0.5)	0	1 (0.8)	0			
**Mean time for changing initial procedure (days, SD)**	20.0 (19.2)	52.2 (21.8)	29.6 (16.3)	64.3 (19.8)	Difference of means: 33.5 days (95% CI 30.6–36.4, p = 0.0001)		
**Treatment outcomes at 6 months** *	**N = 27**	**N = 23**	**N = 38**	**N = 39**			
Cure	20 (74.1)	18 (78.3)	27 (71.1)	31 (79.5)	47 (72.3%) *vs* 49 (79.0%)	0.91 (0.75–1.11)	
Failure	2 (7.4)	2 (8.7)	2 (5.3)	0	4 (6.2%) *vs* 2 (3.2%)	1.91 (0.36–10.6)	
Default	1 (3.7)	0	9 (23.7)	7 (18.0)	10 (15.4%) *vs* 7 11.3%)	1.36 (0.55–3.55)	
Death	3 (11.1)	3 (13.0)	0	1 (2.5)	3 (4.7%) *vs* 4 (6.5%)	0.72 (0.17–3.07)	
Not TB	1 (3.7)	0	0	0			
**Drug Sensitivity Testing** *							
Resistant	4 (14.8)	3 (13.0)	10 26.3)	0	14 (21.5%) *vs* 3 (4.8%)	4.63 (1.34–16.0)	0.01
Sensitive	20 (74.1)	14 (60.9)	27 (71.1)	30 (76.9)	47 (72.3%) *vs* 44 (70.9%)	1.02 (0.82–1.27)	0.87
Not performed	1 (3.7)	4 (17.4)	1 (2.6)	3 (7.7)			
**Patients satisfaction**							
Satisfactory	115(53.7)	117(54.9)	0	0	115 (53.7%) *vs* 117 (54.9%)	0.98 (0.82–1.16)	0.88
Regular	36(16.8)	29(13.6)	0	0			
Unsatisfactory	10(4.7)	10(4.7	0	0			
not Answered	53(24.7)	57(26.7)	0	0			

* % of treatment outcomes and drug sensitivity testing are expressed as a proportion of those who started TB treatment

The mean time for changing the initial prescription was greater in those allocated to LJ at both sites (MD 33.5 days 95% CI 30.6–36.4, p = 0.0001). Only one patient at HUCFF had a positive smear microscopy with a negative culture result (MGIT). The patient was started on treatment before the availability of the test result and did not stop after the negative MGIT result. Doctors considered him to have a clinical diagnosis of TB and a probable cure. The same situation occurred at PAAP for six patients, all of whom were in the LJ group.

No important differences were observed on treatment outcomes in both groups at the end of 6 months. Those participants who had smear microscopy negative at 6 months or completed treatment with clinical improvement were considered cured. One patient did not have TB. We detected 17 cases of drug-resistance. Treatment outcomes of drug resistant TB participants at HUCFF (N = 7), were as follows: 2 cured (single drug resistance), 4 treatment failure and one death; at PAAP (N = 10), 7 cured (single drug resistance), 2 treatment failure and one default.

Severe to moderate adverse reactions to anti-TB drugs occurred at similar rates in both groups and were hepatotoxicity (8) and confusion (4), itch (4), joint pain (11), abdominal pain (7) and nausea/vomiting (3). The participants at HUCFF tended to be equally satisfied with their course of care regardless of which group they were in.

## Discussion

Demonstration studies have shown that liquid medium has high accuracy and provides earlier results than solid medium [6,18–20], and this randomised trial confirms that the mean time for positive results and final TB diagnosis result was shorter using the MGIT960 system.

The results of this study indicate that the use of MGIT960 to detect TB in persons suspected of having the disease represents an advantage over the LJ method in both secondary and tertiary health unit levels in the city of Rio de Janeiro. Despite rates of positive cultures with both methods were not statistically different (16.7% in MGITs vs 14.8% in LJs), the decreased time to positivity with MGIT960 culture led to increased change in management as the majority of patients started anti-TB treatment and only two stopped treatment after test results. These improvements may lead to important outcomes, such as reducing the TB diagnosis delay in patients at increased risk of mortality, especially in those treated at hospitals [21–24].

No clinical or statistically significant difference was observed between the groups assigned to the two diagnostic tests in terms of death at the end of the second month, adverse reaction, bacteriological conversion in the 6th month, or user satisfaction. These outcomes are likely to be affected by other covariates not recorded in the study. Yoon et al., in an observational study, described similar results after evaluating the clinical impact of using molecular testing via Xpert MTB/RIF (Xpert) [25]. They confirmed the high sensitivity and reduced time-test result for Xpert, but did not find lower mortality when compared to the previous practice using smear microscopy and culture tests [25].

As described in a survey of patients suspected of having TB in 539 hospitals in seven participating Asian cities, the proportion of hospitals in which a sputum smear was always performed ranged from 86% to 100%, but the proportion of laboratories performing sputum cultures ranged from 14% to 38% [26]. Sputum cultures were available in a higher proportion of private hospitals, but were rarely used for routine examination of patients suspected of having TB. When evaluating these patients in hospitals in low- and middle-income countries where the occurrence of atypical pulmonary TB, co-morbidities and extrapulmonary TB are more frequent, and where smear microscopy has low sensitivity, it is necessary to rely on culture or molecular methods for rapid differentiation and identification of the mycobacterial infectious agent [21–26].

Liquid medium is considered more effective than solid medium for the diagnosis of nontuberculous mycobacterial [20] and for extrapulmonary TB [27]. We did not find any difference in the identification of atypical mycobacteria isolated from clinical specimens of patients seen in the hospital or at the secondary health unit, but we did find higher proportion of Mycobacterial tuberculosis isolated in extrapulmonary specimens in the hospital. There was also no difference in the culture contamination rate between the liquid and solid medium. These results are similar to those described by Giampaglia et al [28], but differ from those observed by others [29,30]. The low proportion of contamination most likely resulted from the use of liquid medium in a reference laboratory with prior experience.

We observed a low proportion of drug-resistant TB in patients cared for at both health units, and these results match previous drug resistant survey results [31]. It is expected that early access to drug susceptibility testing for first and second-line drugs with MGIT960 would provide a better clinical outcome and prevent the development of extensively drug resistant tuberculosis, as highlighted by others [32]. We have no other explanation than pure chance for having 10 drug-resistant TB cases in the MGIT960 arm and none in the LJ arm at PAAP. It can be noticed that in the MGIT group at PAAP, although 29 participants were smear positive, just 21 started treatment before culture test result. This could be due to the fact that, being an outpatient service, some subjects do not attend the health facility immediately upon being summoned. In the LJ group, since test result is more time consuming, this delay brings no consequences.

### Generalisability

Because this study was designed to inform practice, it was conducted with typical care settings, providers and participants. The study population at both sites can be considered representative of places with similar incidence rates of TB. Thus, these results may reflect many aspects of the Brazilian Unified Health System or regional physician clinical practices, and the generalisability of these findings may be considerable, particularly within Brazil.

### Strengths and limitations

From a clinical point of view, the value of using a new diagnostic test depends on whether it improves patient outcomes beyond those outcomes achieved using the standard diagnostic test already in use. To our knowledge, this is one of the first studies to compare the effects of two alternative TB bacteriological diagnostic tests on TB outcomes. The strength of this study lies in its design, particularly the random allocation of the test and the use of intention-to-treat-analysis.

Although the randomisation process was undertaken at the lab, in this pragmatic approach we could not guarantee blinding of health care workers or patients, but with the exception of “satisfaction with care”, all outcomes were as objective as possible to limit potential ascertainment bias.

We had estimated the sample size of this trial based on a 10% absolute increase in changing the initial prescription within 2 months after randomization, which is an outcome considered important by clinicians. We observed an absolute increase of 6.3%, but the trial was stopped earlier than anticipated, as described above. Nevertheless, our main findings are statistically significant.

Drug resistance was not the focus of our study; therefore, key parameters that would help evaluate the clinical impact of the use of MGIT 960 in patients suspected of having drug-resistant TB were not examined.

## Conclusion

We assumed that the selection of an effective test for TB is just as essential as the selection of effective treatments. The results of this trial suggest that using MGIT960 for the diagnosis of drug-susceptible TB provides an effective model for TB control at both the secondary and tertiary levels. This randomised pragmatic trial also illustrates how the objective evaluation of new diagnostic tests can be undertaken worldwide.

## Supporting Information

S1 Fig CONSORT flow diagram for study participants. (PDF)

S1 Text Protocol—English version. (DOCX)

S2 Text Protocol—Portuguese version. (DOC)

S3 Text CONSORT checklist. (DOC)
