# Supplementary figures and images for: Liquid vs Solid Culture Medium to Evaluate Proportion and Time to Change in Management of Suspects of Tuberculosis—A Pragmatic Randomized Trial in Secondary and Tertiary Health Care Units in Brazil
Source: PLoS One. 2015 Jun 5;10(6):e0127588. doi: 10.1371/journal.pone.0127588 (PMC4457845; doi:10.1371/journal.pone.0127588)

S1 Fig. CONSORT flow diagram for study participants

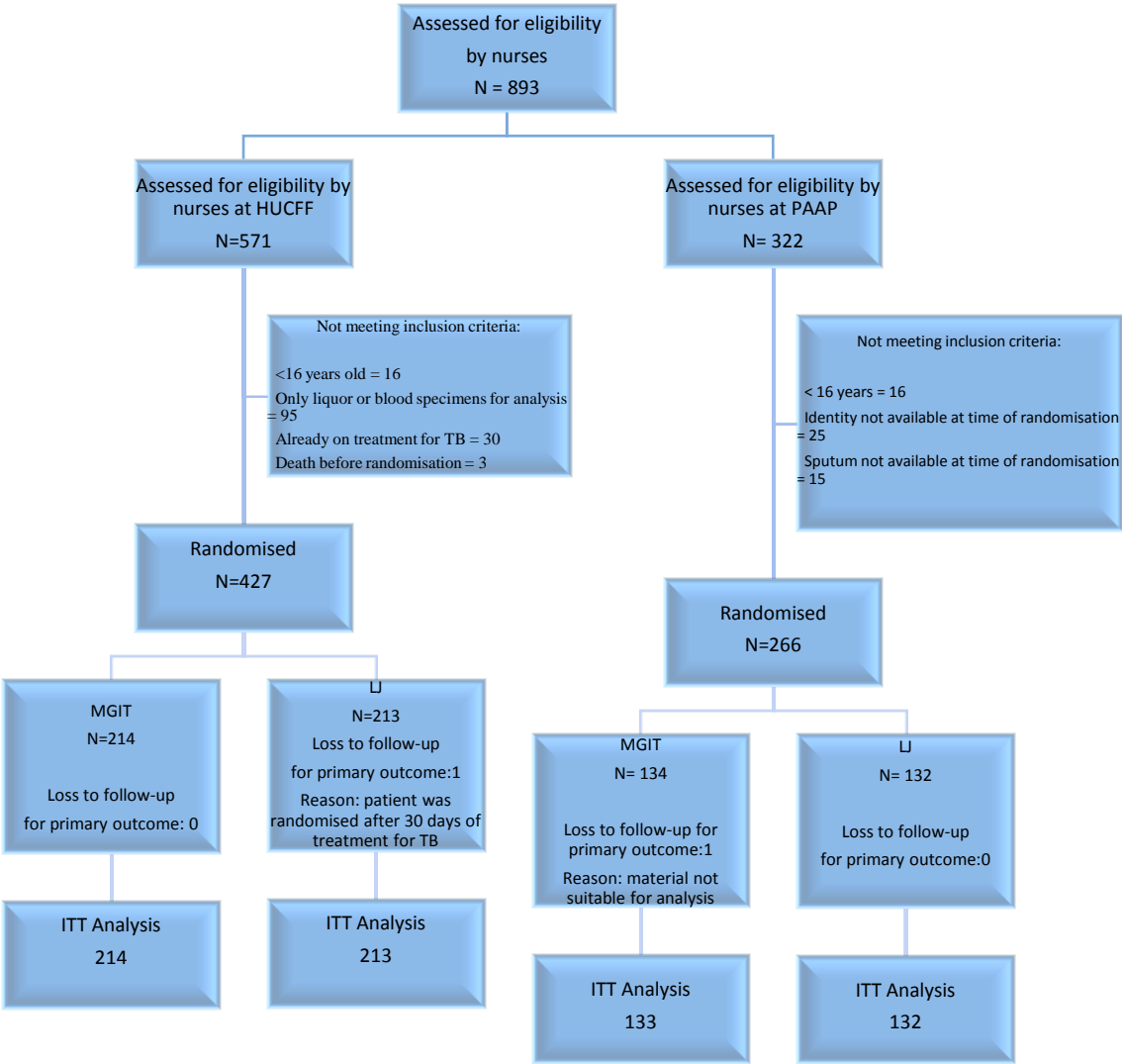

Supplement: S1 Fig — (PDF) [file pone.0127588.s001.pdf]
