# Supplementary material for: Liquid vs Solid Culture Medium to Evaluate Proportion and Time to Change in Management of Suspects of Tuberculosis—A Pragmatic Randomized Trial in Secondary and Tertiary Health Care Units in Brazil
Source: PLoS One. 2015 Jun 5;10(6):e0127588. doi: 10.1371/journal.pone.0127588 (PMC4457845; doi:10.1371/journal.pone.0127588)
Supplement: S1 Text — (DOCX) [file pone.0127588.s002.docx]

**Pragmatic Clinical Trial of techniques for the Tuberculosis diagnosis in patients assisted at health Unit in Rio de Janeiro, Brazil**

**Principal Investigator**

Prof. Dr. Afranio Kritski

Research Unit Coordinator - FM/IDT/HUCFF-UFRJ

Coordinator of the REDE-TB - Diagnostic Area

**Institutions that participate in the study**

1. TB Academic program of the FM/IDT/HUCFF-UFRJ/ Rede-TB - Diagnostic Area

**Managing Committee Responsible for Planning and Conducting the Clinical Trial**

Dr Afrânio Kritski - UFRJ’s Chest Physician and epidemiologist - Coordinator of the study

Dr. Gisele Huf - Clinical trial expert - INCQS - FioCruz

Dr. Leila Souza Fonseca- UFRJ’s Microbiologist - President of the Managing Committee

**Study Monitoring Group**

Prof. Jose Roberto Lapa e Silva - Professor of Pneumology of UFRJ.

Profa Fernanda Mello – Associate Professor of Medical School of UFRJ

**Work Group**

Dr. Fábio Aguiar – Physician at TB Control Program

Ms Adriana Rezende – Research Nurse

Ms Anna Grazia Marsico – Biologist and Chief of Mycobacteriology Laboratory

**Protocol Synopsis**

The rapid TB diagnosis is essential for decreasing the TB transmission and so the number of TB infected individuals. The bacilloscopy has low sensitivity and the major problem of the culture for mycobacteria is the long time of results realeasing. In spite of the costs, new tests can reinforce the Hospital Program of TB Control. The studies on MGIT accuracy have showed similar results with those obtained with the gold standard – culture on solid medium – as on the *M.tuberculosis* isolation as on susceptibility test to major drug for TB treatment. Using a pragmatic clinical trial design this study intends to evaluate the effectiveness of MGIT test versus routine culture methods in two university hospitals.

**Eligible Patients**

Individuals aged 16 or more, assisted in university hospital and secondary health unit in Rio de Janeiro, Brazil, under tuberculosis suspicion (TB), with or without anti-TB treatment in the past.

**Excluded patients**

Individuals that do not sign the Term of Free and Clear Consent (or their legal representative) or when there is a refusal of participation. Patients with known active TB

**Study Planning:** Pragmatic, open and randomized clinical trial to be accomplished in two university hospitals in the southeast area of Brazil.

**Intervention**

After the randomization, TB suspects will be submitted to MGIT or culture in solid Löwenstein-Jensen medium (gold standard)

**Main objective:**

1. The primary outcome was defined as the proportion of patients in each group whose initial prescription changed by the second month after randomization. The ‘change’ could be initiating or stopping TB treatment.

**1) RATIONALE**

The rapid diagnosis of TB is important in order to prevent the disease. However, little progress has been done in the last 50 years. New techniques such as the amplification of nucleic acids and automated systems using culture in liquid medium are very expensive and depend on sophisticated techniques that make them inadequate for routine use in "low income countries." Yet, even in "middle income countries" as in Brazil, where such technologies could be used in the public system and TB is endemic, the TB diagnosis has been based only on clinical suspicion, chest radiography and bacilloscopy exam (BAAR) of the sputum. Despite the low sensitivity (40% to 60%) of the sputum bacilloscopy, it is still one of the most important tests for the TB diagnosis, as it detects the patients that present a higher chance of infecting the community. Besides, in the developing countries a low incidence of the non tuberculosis mycobacterium (NTM) disease is observed. In these areas, the sputum bacilloscopy has a highly positive predictive value. However, in most of these countries, in practice, the culture for mycobacteria, whose sensitivity is higher (80% to 85%) than the bacilloscopy in the diagnosis of the lung TB, is carried out in solid medium, Löewenstein-Jensen (LJ), and is only performed in selected clinical cases (cases of treatment failure, patients with negative bacilloscopy). The major problem of the LJ culture is its long time of incubation (4 to 6 weeks) and the sensitivity test is accomplished from the culture and not from the clinical specimen, which demands several additional weeks for the obtainment of the results. In practice, in these countries, with the universal use solely of the sputum bacilloscopy, about 30% to 40% of the patients assisted in Primary Health Units in developing countries such as Brazil are treated of TB without bacteriological confirmation.

Brazil has the second largest number of TB cases in Latin America, with approximately 92.000 yearly new cases and the thirteenth largest number of cases in the world. The largest incidence rate of drug resistant TB (DR- TB) and of TB associated to HIV (TB-HIV) occurs in the metropolitan areas of Vitoria, Rio de Janeiro and Porto Alegre. The analysis of the preliminary data described in the national literature, the primary multi-resistant TB rate (MDR-TB) varies from 1 to 3% in the Primary Health Units (UPS) and from 5 to 12% in the Hospital Units, being higher among HIV+ patients. The highest TB lethality rate occurs due to the incorrect diagnosis accomplished in Hospital Units, Emergency Units, usually without TB control activities. In these hospitals, only 30% of the bacteriology laboratories perform culture tests for mycobacterium and they seldom make available the result of the TB bacillus sensitivity test to drugs. It is considered that in 20% to 30% of these cases the TB diagnosis was incorrect and the inadequate treatment probability is still higher in areas or in Health Units with a more extensive assistance to HIV infected patients or co-morbidity bearers, as in Hospital Units.

In 2004, it was emphasized in the Guidelines of National Norms/Policies of the Ministry of Health the need to introduce the method of culture for mycobacterium in an universal way in areas with high HIV infection rate and sensitivity to drugs tests (TSD) in reference Health Services or that presented high prevalence of DR-TB, as in hospitals, prisons, etc.

With the aim of stopping the global increase of TB, the WHO has recommended the DOTS strategy since 1993. This strategy is based on the diagnosis of pulmonary TB through sputum bacilloscopy and the shortened treatment schedule containing rifampicin (RIF), isoniazid (INH), pyrazinamide (PZA) and ethambutol (EMB). Yet, even with the DOTS strategy adoption in the areas or in Health Units where the MDR-TB is highly prevalent, the use of the first line schedule treatment (INH, RIF, PZA and EMB) is not effective and is contributing to the increase of primary MDR-TB in several areas of the world. In Russia, MDR-TB was initially prevalent only in patients with TB in the prisons, but recently, in these areas, the primary MDR-TB is occurring in patients assisted in Hospital Units or in Primary Health Units (UPS). Studies with mathematical models show that 75% of the MDR-TB cases need to be detected and 80% of them must present a favourable result so that the incidence of new MDR-TB cases is reduced in these areas. With the MDR-TB treatment, even with standardized schedules as the ones available in Brazil, only 60% to 70% of the patients obtain favourable results with the treatment. The rapid diagnosis of almost all of the MDR-TB cases has, therefore, become essential for the success of the program in these places.

The *M*. *tuberculosis* sensitivity to drugs determination consists basically in modifications of the minimum inhibiting concentration method in solid medium culture (proportions, resistance reason and absolute concentrations) and in liquid medium culture (BACTEC 460TB)*.* The methods carried out in solid medium culture, although quite reproducible, are very arduous, need great biosafety strictness and their results are given after a long period of time (15 to 30 days). The method validated in the Bactec 460TB system (liquid medium culture), in spite of being operationally simple and providing a rapid result, is a method whose radiometric principle drastically restricts its use. Besides those aspects, it has to be mentioned that the identification of the *M. tuberculosis* compound*,* obtained by phenotypic or genetic analyses, is a mandatory prerequisite for the accomplishment of the drug sensitivity test*.* The existent identification tests are fully reliable and of low cost. Nevertheless, they do not provide a swift result. In these situations, the MDR-TB bearer patients (mainly those in hospitals, infected by HIV and/or co-morbidity bearers) have frequently used the inappropriate schedule and/or developed to death before medical measures were taken.

In the last years, in the United States of America (USA), the *Center for Disease Control* (CDC) has recommended the use of the MGIT system (Mycobacteria Growth Indicator Tube) developed by the same company that developed the BACTEC 460 TB system (Becton Dickinson Diagnostic Instruments Systems, Sparks, Md). That is a non-radiometric method that contains the Middlebrook 7H9 modified liquid medium and is based on the same principle of the MGIT system, but is a totally automated system that presents continuous monitoring equipment, capable of 960 simultaneous tests. The instruments collect the fluorescence data of each tube at every 60 minutes and when an increase of this fluorescence is detected, the instruments indicate the tube as positive.

The development of new products for the tuberculosis diagnosis faces some challenges, the same way it is observed in the development of new medicines for neglected diseases: scarce financial resources and the market logic that reduces considerably the interests of large companies, since the use of the final product will be almost totally accomplished by the government of the different countries, usually not fulfilling their contracts, exactly where such diseases are prevalent.

In the last years, it has been emphasized the necessity of the accomplishment of Phase IV clinical trials, called pragmatic, which assess interventions in the nearest possible way to the real conditions in which they are applied in the clinical practice, serving as a support for taking decisions, in opposition to the so called explanatory Phase I, II, III clinical trials, carried out in more controlled conditions, answering questions about if and how does an intervention work; these questions are necessary to obtain the registration and commercialization of products.

Pragmatic studies, for being developed in routine conditions, do not complicate the usual practice and for this reason they are cheap and get the voluntary collaboration of the team.

The attainment of such studies does not raise great interest from the industries and unfortunately their execution usually is not considered a priority on the part of the governments be it at federal, state or municipal level or by the public policies formulators.

Studies already accomplished have shown that the BACTEC MGIT 960 performance is equalled to the BACTEC 460 one, with the advantage of being a non-radiometric method, supplying results in less time than the LJ solid medium. Recently, the Food and Drug Administration (FDA) has approved the automated method that uses the liquid medium called BACTEC MGIT (Mycobacteria Growth Indicator Tube) 960 that besides being as non-radiometric as the previous version (BACTEC460), provides a reliable TB and DST diagnostic result in less time than the LJ solid medium (10 to 15 days).

Usually, when the diagnostic kits are validated by the US FDA or by the European regulatory organizations, the regulatory organizations of developing countries tend to provide the registration for commercialization. In the last years, though, in Brazil, researchers of the Brazilian Net of TB Research (REDE-TB) have suggested that before releasing the registration and its allocation in the national market, the new diagnostic methods, mainly the ones related to the public health impact diseases should be submitted to an evaluation of their effectiveness and cost-effectiveness relation, in routine conditions, through pragmatic clinical trials.

Since there is no report in the literature of studies about pragmatic clinical trials and the cost-effectiveness of new technologies for the drug sensitive tuberculosis (DS-TB) and DR-TB diagnosis in Hospital Units of emerging countries, the Becton and Dickinson Company sought the REDE-TB in 2002 and proposed a partnership in this area. Recently, a Bactec MGIT 960 inter-laboratorial validation study has been concluded in reference laboratories in the cities of Rio de Janeiro, São Paulo and Vitória, with excellent results (Giampaglia et al, Multicentre evaluation of automated BACTEC 960 system for susceptibility testing of Mycobacterium tuberculosis. [Int J Tuberc Lung Dis](javascript:AL_get(this,%20'jour',%20'Int%20J%20Tuberc%20Lung%20Dis.');). 2007; 11: 986-991). Following a strategic planning, there has been agreed on the accomplishment of a study on phase III/IV of this technique in hospital or Primary Health Units with different TB/HIV rates. The company took the responsibility in supplying the inputs. In the present project, the evaluation of the clinical impact and cost-effectiveness relation of a new TB diagnostic technique is intended. This will take place in the same Health Units in which the Diagnostic Clinical Trials (accuracy analysis in routine conditions) are being carried out by REDE-TB researchers.

**2.- Hypothesis.** The use of a new technology (BD960) for the TB, MDR/TB investigation in secondary health and hospital units, besides reducing the intra-hospital TB transmission and the cost of the hospitalized patient's treatment, will also greatly reduce the morbid/lethality of TB patients, as they are usually bearers of other co-morbidities, mainly the HIV infection or the ones that use immunosuppressant chemotherapy. With the use of the current diagnostic investigation routine in the participating health units (method of proportions), the drug resistance diagnosis is usually available too late, when adverse effects and/or death have already occurred.

3. - **METHODOLOGY DETAILING**

**3.1 - Location of the Study**

1. Hospital Complex: Clementino Fraga Filho University Hospital and Chest Diseases Institute of the Federal University of Rio de Janeiro (HUCFF-IDT/UFRJ), located in the city of Rio de Janeiro.

2. Policlínica Augusto Amaral Peixoto (PAAP) – Municipality Health Secretary - Rio de Janeiro, Brazil.

**3.2 - Selection of Patients / Ethical Aspects**

Patients will be considered eligible in the participating health units with TB suspicion. Written consent will be obtained from all participants. The verbal consent with the signature of a witness will be obtained for individuals unable to read. The individuals will be informed that the study is being brought about to determine the effectiveness of a new diagnostic test for TB. For the candidates that choose not to be included in the trial, the standard diagnostic investigation will be offered, characterizing them as not participants of the study. The consent process will include the information provision regarding the risks and benefits of the drugs of the study.

**3.3 Inclusion Criteria**

a) age > 16 years; b) with clinical and suggestive laboratorial data of the first episode or active TB;

**3.4. Exclusion Criterion**

a) Patients that do not sign the Term of Free and Clear Consent (or his/her legal representative) or refuse to participate; b) Patients under anti-TB treatment; c) history of culture positive for M tuberculosis in the last 6 months prior of randomization.

**3.5. Interventions**

After randomization, clinical specimens from TB suspects will be submitted to MGIT or culture in Löwenstein Jensen solid medium (gold standard) at the HUCCF-UFRJ Mycobacteriological Laboratory. The accomplishment of such tests does not incur in risk for the patient.

**3.5.1. Description of the tests**

Respiratory and non-respiratory specimens, naturally contaminated, will be collected in disposable receptacle. The specimens should be given to the laboratory up to 24 hours after being collected and will be submitted to bacilloscopy after the Ziehl-Neelsen coloration. After being processed by the Kubica method, they will be cultivated by the BACTEC MGIT 960 method and in Löwenstein-Jensen in Rio de Janeiro and in Vitoria, and by the BACTEC MGIT 960 method. Cultures contaminated by other microorganisms will be called as contaminated and considered as negative for calculation in statistical tests. In the clinical specimens of the included patients, the sensitivity test will be carried out by the gold pattern tests and by BACTEC MGIT 960. Additionally, the positive cultures will be appraised as for the sensitivity of *M. tuberculosis* for isoniazid, rifampicin, streptomycin and ethambutol by the indirect test of proportions (one strain/patient).

***M*. *tuberculosis* Identification*:***

Conventional methods - The selected strains will be identified as *M. tuberculosis,* based upon the characteristics of the colonies (rough, cream and opaque), in the capability of producing niacin, nitrate reductase and catalase heat inactivation.

**Identification of the *M. tuberculosis* growth by the BACTEC MGIT 960 System** - In the bottom of each tube there is a fluorescence sensor. The fluorescence level that each tube emits corresponds to the amount of oxygen consumed by the organism, which is proportional to the number of bacteria in the tube. When the oxygen is consumed, an oxi-reduction reaction occurs causing the increase of fluorescence. That fluorescence can be detected through a transiluminator with a UV bulb in a 365 nm wavelength. Five hundred microliters of treated sediment of the clinical specimens will be inoculated in each one of the MGIT tubes without and with PNB in a 500g/mL concentration. The result of the *M*. *tuberculosis* growth identification will be defined based on its growth inhibition in the MGIT culture medium containing PNB and the growth (positive) in the MGIT control tube.

*A standardized suspension of the M. tuberculosis, M. bovis, M. africanum strains and of other species of mycobacteria grown in solid medium will be prepared. Five hundred microliters of the suspensions will be inoculated in MGIT 960 medium with 500mg/L of PNB and control medium without PNB. The flasks will be homogenized, identified through bar code reading and placed into the specific shelf-carrier of the BACTEC MGIT 960 instrument. The result of the M. tuberculosis growth identification will be defined based on its growth inhibition in the MGIT culture medium containing PNB and the growth (positive) in the MGIT control tube. The result is given between 5 to 15 days, depending on the bacterial growth.*

**Drug sensitivity test with the BACTEC MGIT 960 System -** The *M. tuberculosis* strains will be subcultivated in MGIT tube until it indicates a positive result in the BACTEC MGIT 960 System. Five tubes of MGIT (7 mL) will be identified and placed in the shelf-carrier (AST Set Carrier) in the following sequence: GC (control - Without drugs), STR (Streptomycin), INH (Isoniazid), RIF (Rifampicin) and EMB (Ethambutol). Then, a volume corresponding to 0,8 mL of BACTEC MGIT SIRE Supplement will be added to each tube. Aseptically, using a micropipette, 100 μl will be pipetted of the stock solution of each drug in the 4 tubes, so as to reach the final concentrations presented in table 1. Starting from the *M. tuberculosis* cultures with growth in the MGIT tubes a 1:100 dilution will be prepared, by the addition of 0,1 mL of the cultures in 10 mL of sterile saline solution. After the homogenization of the bacterial suspensions, 0,5 mL of the diluted suspension (1:100) will be inoculated in a tube of MGIT culture medium (control without drugs). In other the tubes containing the drugs (INH, RMP, EMB and SM) 0,5 mL of the not diluted suspension will be inoculated. The flasks will be homogenized, identified through bar code reading and placed in the shelf-carrier of the sensitivity test in the BACTEC MGIT 960 instrument. The results (sensitivity or resistance to the drugs) shall be defined according to the criteria established by the manufacturer (Becton Dickinson), based on the algorithm of the System computer program.

**Drugs sensitivity test by the method of the proportions -** It will be carried out based on the indirect method described by Canetti, Rist and Grosset (1963). Starting from the bacterial growth in solid medium, without drugs, a suspension will be made, using the largest number of colonies possible, in a 16 x 150mm tube containing about 10 glass pearls and approximately 0,5 mL of sterile distilled water. The tube will be agitated in vortex for 20 to 30 seconds in order to homogenize the suspension. Then it will be slowly dripped into the sterile distilled water suspension until a turbidity is obtained, corresponding to tube number 1 in Mac Farland's scale or to a BCG 1mg/mL standard suspension. From this standardized suspension decimal dilutions will be made until 10^-6^ and 0,1mL will be inoculated in the control tubes without drugs and in the tubes with drugs. After the inoculation, the tubes will be placed horizontally inclined on a tray so that the inoculum is distributed on the whole surface of the medium, keeping them with the lid loose for 24-48 hours in order to dry the inoculum. The tubes will then be incubated in vertical position at 37ºC for 28 days. After this period, the reading of the test and the results interpretation will be accomplished according to the recommendations of the Bacteriological Norms of the Ministry of Health Technical Manual.

In HUCCF-UFRJ Mycobacteriological Laboratory, the several tasks are distributed among the technicians. Thus, a technician is responsible for the daily processing of the clinical specimens through the Kubica method and for inoculation the sediment in Lowenstein-Jensen medium. Another technician is responsible for carrying out the drugs sensitivity tests through the method of proportions, a third one for the inoculation and sensitivity test in the tubes of the BACTEC MGIT 960 method, of its specimens and of other studies, etc. This way, there is no possibility for the technician to be induced to favour the positive reading of one or another method. To this division of tasks is added the fact that the MGIT960 reading is totally automated, the result being printed by the computer of the equipment in thermal impression ribbon, which will be photocopied and filed in a suitable place.

**3.6. Randomization - Clinical procedures for Open Clinical Trial Practice**

The physician that routinely evaluates the patients under active TB suspicion, invited to voluntarily participate of the study, once deciding the patient's inclusion in the protocol will request an envelope that contains in its external side four key questions: a) which is the patient's probability to present TB (high/intermediary or low); b) the choice above was based in the results of radiological examination; c) in his/her opinion, should the patient begin anti-TB treatment (yes or not) d) what is the patient's risk of having resistant TB (high/intermediary or low). The four questions filled out, the doctor will open the envelope and inside it he/she will identify which is the diagnostic method that should be requested for the patient. The envelopes will be numbered in growing order and the indication of the diagnostic tests inside them has been previously randomized by a professional non-participant of the study. This procedure **attempts to guarantee the concealing of the randomization and at the same time it will be possible to check the success of the randomization**.

In the routine investigation of patients under TB suspicion, the following information is collected in standardized forms: a) socio-demographic data; b) organs and human body systems examination; c) exposure to TB, disease and treatment history; d) past pathological history; e) physical exam; f) chest X-ray result; g) tuberculin skin test result; h) HIV test result.

Respiratory specimens include spontaneous sputum, induced sputum and bronchoalveolar wash. Non-respiratory specimens include biopsy material and synovial, pleural, CSF liquids, etc. A minimum of two and a maximum of three specimens per patient will be appraised in this study. Clinical specimens will not be collected out of the TB diagnostic investigation routine in the hospital.

Following the routine, the clinical samples will be forwarded to the mycobacteriology laboratories in the next working day. Only in the clinical samples of patients that have received randomization for the MGIT, should the laboratory personnel be informed that the MGIT must be performed.

To analyze the outcome of the study, the key information below, present in the medical handbooks or routine record cards of the TB Program, will be copied to the study’s own forms.

**OUTCOMES, INDICATORS AND RESPONSIBLE FOR THE DATA COLLECTION**

| Outcome | Indicator | Responsible for the information collection |
| --- | --- | --- |
| 1. Time in hospital | PCTH Record card | Nurse |
| 2. Number of deaths | Related toTB  Black book, PCTH-Amb Record card, Medical handbook  Related to non-TB  HUCFF’s Medical handbook | Nurse |
| 3. Result of the treatment  (cure, abandonment, treatment failure, change of diagnosis) | Black book , PCTH Record card – Ambulatory | Adriana |
| 4. N general adverse effects related to drugs | Medical handbook | Physician |
| 5. N adverse effects related to anti-TB drugs | Medical handbook, PCTH Record card | Physician |
| 6. N adverse effects related to the use of diagnostic methods (i.e.: bronchoscopy, biopsy, induced sputum, etc) | Medical handbook | Physician |
| 7. Time of the diagnosis (in days)  From the randomization to the result release by the Lab | Book of the laboratory - or electronic data in Medtrek | Nurse |
| 8. Time of sensitivity Test | Lab Book / Medtrek | Nurse |
| 9. Time of anti-TB schedule change | PCTH Record card at the end of the 2^nd^ month  PCTH Record card at the end of 6^th^ month | Nurse |
| 10. Proportion of patients with false negative bacteriological result | Medical consultation at the end of the 2^nd^ month | Physician |
| 11. Clinical Worsening | Medical handbook / PCTH Record card 7, 15 30, 60, 180 days | Physician |
| 12. Radiological Worsening | 60 days | Physician |
| 13. Time of symptoms and beginning of the anti-TB treatment | Medical handbook / PCTH  PCTH Record card - Ambulatory and Active Search | Nurse |
| 14. N processed materials until the TB diagnosis | Card registry of the laboratory | Nurse |
| 15. N processed materials after the TB diagnosis | Card registry of the laboratory | Nurse |
| 16. Proportion of patients that develop resistant TB | Laboratory Book - PCTH Record card | Nurse |
| 17. Proportion of bacteriological conversion at the end of the 2^nd^ month | Laboratory Book , Medical Handbook, PCTH Record card | Nurse |

Other information, necessary to analyze the outcomes related to the cost- effectiveness analysis that are not available in the medical handbooks or routine record cards of the Hospital Program will be collected in a standardized way (in annex) by a professional trained at SIA-SUS and in other database of the Ministry of Health and Ministry of Education.

Study forms

- Forms of free and clear consent and of inclusion
- Eligibility forms for selection
- Data collection forms that document the inclusion, the follow-up visits and the specimens collection in the weeks 8 for patients under diagnostic investigation and in the weeks 8, 24 for patients that receive the diagnosis of activeTB.
- Laboratorial form that documents the receiving, processing and results of the clinical specimen.
- Administrative forms that document the participants' deaths, end of the study drugs, direction and adverse events treatment and treatment failures.

**3.6. Data obtainment**

All of the forms will be created in Word, and the data will be included in the ACCESS database. All the data will be filled out according to the instructions that will be defined and detailed in a specific manual. The patients' names shall be collected only once and this information will be kept separately in a form that does not contain clinical or laboratorial data. The number of the patient's identification will be used in all of the forms that contain results. The evaluation of the clinical data quality will be accomplished twice a year, and 10% of the patients included in the 6 previous months will have all the primary and computerized results revised.

**3.6.1.-Storage and filing of the registrations**

The director of the study, Dr. Kritski, and the data manager will be responsible for keeping the storage of all of the used source registries and to fill out the forms of the study data survey. The source registries may include requests and laboratorial reports, clinical notes and documentation of the references. All the forms of the study data survey and source registries will be stored in files locked in a safe area. All the information of the locater will be maintained in locked rooms in a safe area and separate from all the other documents of the study. The access to the data files and the study registries will be limited to the study personnel.

**3.6.2 - Data handling**

An original ID number of the study will be attributed to each participant. This number will be registered in each data form and clinical specimen to facilitate the data network. Names and other obvious identifiers shall not be used in forms, and will only be used in the clinical specimens that are collected during non-programmed visits of the clinic for immediate care. If necessary, the questionnaires and the specimens will be identified by a two digit number of visit, by a one digit code for type specimen, and by a one digit code that identifies the aliquot of the specimen for deposited specimens of the sputum. To minimize transcription mistakes and to assure that each instrument of the data survey and each specimen will be correctly labeled, the pre-printed labels with the ID number and the participant's name will be generated to label each form and specimen.

The manager of the place will keep follow-up books to register the dates of the clinic visits made, the ones to come, as well as the dates of specimens collection. In addition, for each patient a study file will be kept, containing all the forms, laboratory results and other information pertinent to the study. All of the follow-up books and registrations will be kept in filing closets, locked when not in use and accessible only for the clinical personnel.

The laboratory tests will be requested in the requisition forms of the study’s test laboratory that are printed on a pressure sensitive paper, of multiple sheets. In the laboratory, the test results will be registered on the requisition form and the sheets will be separated and distributed as follows: the bottom sheet stays at the laboratory, the middle sheet is returned to the clinic and the top sheet goes to the computer room for data insertion.

**All the finished questionnaires will be collected daily from the clinic and introduced into the computer within 72 hours.** An adapted system of double entry will be used to computerize all of the study instruments. The forms received at the computer room will be received under receipt. The forms will be incorporated and verified, and the date of these procedures will be registered on the reception sheet. The data files will be verified so as to see if there are logical and scale mistakes using adapted SPSS programs.

The identification number (ID), the collection date, the specimen volume and the position in the freezer will be registered on the requisition forms of the laboratory and introduced into the computer for all the deposited specimens. The computer files of these forms will be used to create specimen lists for each box of specimens. Two years after the conclusion of the study, the identifiers, excluding the ID number of the study, will be suppressed from the computerized data files and from the paper files.

**3.7. The patient's evaluation during the diagnostic investigation phase (total of 8 weeks) and anti-TB treatment (total of 24 weeks)**

All the patients will be clinically evaluated and continually monitored during the first 8 weeks of the diagnostic investigation phase. Those who receive an active TB diagnosis will be monthly monitored until the end of the drug treatment. The information below, collected in the routine follow-up of the Hospital Tuberculosis Program, will be obtained in the weeks 2, 4, 8, and 24 will be registered in the forms of the study:

▪ Weight

▪ Signs and symptoms associated with TB (e.g. cough, fever, sweating, weight loss, haemoptysis)

▪ Signs and symptoms associated with adverse effects of the drugs of the study (e.g. cutaneous rash, dizziness, fatigue, weakness, anorexia, nauseas, vomiting, diarrhea, abdominal flabbiness, jaundice, neuropathy, palpitations)

▪ If a patient develops new signs or of worsening and symptoms of pulmonary TB, a new chest teleradiography will be performed and three sputum specimens shall be obtained for TB culture and sensitivity tests. If a patient develops signs and symptoms of extra-pulmonary TB, cultures from the appropriate places of the body will be obtained.

Monthly evaluations in the weeks 4 and 24 will include the above indicated procedures and:

▪ A complete hemogram (blood count) with platelet count, creatinine, ALT, AST, Gamma GT, total bilirubin and fractions and alkaline phosphatase

▪ Sputum specimens (2) for direct bacilloscopy (Baar research) and culture for mycobacterium

▪ If a patient is unable of producing sputum naturally, the sputum shall be induced by hypertonic saline solution inhalation

▪ A chest radiography performed in week 8

▪ Drugs sensitivity test accomplished in weeks 8 and 24

In patients that receive active TB diagnosis, hepatitis related to drugs monitoring will be accomplished according to the patterns practiced in TB Control Program. Patients that present suggestive symptoms of adverse reactions to drugs will be examined by the doctor and laboratory tests will be carried out, as suitable, including tests of liver function. The physicians of the study will evaluate each case individually to define the relation of the adverse events to the medications of the study. Classification of possible adverse reactions will be made according to NIAID DAIDS *Severity Grading Schema of Adult Adverse Experiences*. The patients diagnosed with possible Grade 3 toxicities related to the drugs, will have the medication that is more likely associated with the adverse event temporarily discontinued. The medication will be resumed when the toxicity is solved. Patients with Grade 4 toxicities will have the medications of the study that are more likely associated with the adverse event permanently discontinued.

Patients considered as in failure of anti-TB treatment shall be treated to the assisting doctor's criterion, according to TB Program routine for these cases. The patients suspicious of treatment failure will be evaluated as for the emergency of TB resistant to drugs and will continue to be followed up clinically and laboratorial according to the protocol agenda until the end of the study.

The participants whose initial culture presented *M*.*tuberculosis* resistant to any of the drugs of the study will be treated with a regimen individually tailored for the susceptibility pattern of the isolated one, as established by the doctors of the study.

**Concomitant medications during the study phase**

The use of any drug (including medications of current use and not controlled) during the initial phase of diagnostic investigation and anti-TB therapy will be monitored and registered on the Concomitant Medication Form. The concomitant antimicrobials with known antituberculosis activity (for instance, rifabutin, rifapentin, amoxicilin/clavulanic acid, amicacin, canamicin, streptomycin, ofloxacin, ciprofloxacin, levofloxacin, sparfloxacin, gatifloxacin, gemifloxacin, linezolid, capreomycin, clofazimine, cycloserine, ethionamide, sodium para-aminosalicylate) will be registered for an analysis *a posteriori* of the final diagnosis. Every patient that receives more than 4 consecutive days of some of the above listed medicines will be classified as being in a regimen that may potentially alter the clinical evolution and his/her inclusion as a non-TB case. Antimicrobials with no anti-tuberculous activity known can be prescribed for intercurrent infections at the investigator’s criterion.

**3.8. Definition of a TB Suspect Case, Active TB Case and Discordance Analysis**

**Pulmonary and /or pleural pulmonary form**

Every patient under pulmonary TB suspicion that presents respiratory signs/symptoms and with alterations in the chest X-ray will be classified in three groups;

a) Typical pattern of TB: infiltrated in upper third (LS or apical seg of LI), with or without cavity; bilateral miliar infiltration; unilateral pleural hemorrhage with upper lobe infiltration .

b) Intermediate pattern of TB: infiltrated in middle or lower third, with or without cavity in these areas; isolated pleural hemorrhage, infiltrated in upper thirds of undetermined activity; diffuse abnormalities (not miliar); mediastinal or hilar enlargement .

c) Atypical Pattern of active TB: multifocal hypotransparency images; alveolar hypotransparency images; calcified nodules.

“**Confirmed TB case"** will be defined as the patient with at least one positive culture for *M. tuberculosis* in L-J or MGIT960*.*

**“Probable TB case"** will be defined as the patient that presents negative results to culture with one or more of the following characteristics:

a) clinical answer to the treatment (defined as improvement to X-ray, b) decrease of the cough and 3% weight gain in relation to the beginning of the treatment) and absence of alternative diagnosis;

Patient not classified as confirmed case or probable TB will be considered "non- TB."

**Treatment failure:** patient that remains with positive bacteriological test (two samples in a 30 day interval) after 5 months of treatment, standardized by MH

High risk factors for resistant TB:

a) Relapsing TB (previous complete treatment for TB with apparent cure), b) Treatment failure (patient under treatment ≥ 4 months with positive bacilloscopy), c) Patient’s contact with multidrug resistant-TB.

3.9 **Main outcome**  The primary outcome was defined as the proportion of patients in each group whose initial prescription changed by the second month after randomization. The ‘change’ could be initiating or stopping TB treatment.

**Secondary outcomes-**

-The secondary outcomes were the mean time to changing the initial clinical prescription,

-The proportion of patients with positive culture and positive smear microscopy for TB,

-The proportion of patients initiating treatment before test results,

-The occurrence of severe adverse events

-Patient satisfaction with the overall treatment- Number of deaths by any cause in the patients included in the study.

- Proportion of patients with TB diagnosis sensitive or resistant to anti-TB medicines (RIF, INH, SM and EMB), with the methods employed in the study.

- Proportion of patients that undergo anti-TB treatment failure during the therapy.

- Proportion of drug resistant TB development in the patients that used the different diagnostic methods and that were classified as treatment failures.

- Proportion of bacteriological conversion (bacilloscopy and culture for mycobacterium) due to the anti-TB treatment at the end of the second and sixth month of anti-TB treatment.

**3.10. Size Sample**

Concerning the primary outcome, the smallest absolute difference considered important by a panel of specialists working at HUCFF was 10% presents the number of patients required to have an 80% chance of detecting this difference at the 5% level of statistical significance. We intended to recruit at least 770 participants.

**3.11 -. Data analysis**

Socio-demographic and clinical characteristics between groups at trial entry will compared. For dichotomous outcomes, we will calculate the proportions for both groups, and for continuous outcomes, we will calculate the means, standard deviations and medians. We will used intention-to-treat analysis to calculate the relative risk, the risk difference, the number needed to treat and the 95% confidence intervals for these parameters. No subgroup analysis will be pre-specified. All analyses will be performed using SPSS software
