# Supplementary material for: Liquid vs Solid Culture Medium to Evaluate Proportion and Time to Change in Management of Suspects of Tuberculosis—A Pragmatic Randomized Trial in Secondary and Tertiary Health Care Units in Brazil
Source: PLoS One. 2015 Jun 5;10(6):e0127588. doi: 10.1371/journal.pone.0127588 (PMC4457845; doi:10.1371/journal.pone.0127588)
Supplement: S2 Text — (DOC) [file pone.0127588.s003.doc]

**Edital MCT/CNPq/CT-Saúde/MS/SCTIE/DECIT**

**Nº 067/2009**

**Apoiar projetos de pesquisa científica e tecnológica para fortalecimento da Rede Brasileira de Avaliação de**

**Tecnologias em Saúde – REBRATS**

**II.1.1.1. LINHAS DE APOIO**

**A - DOENÇAS INFECCIOSAS E PARASITÁRIAS**

**A.10) Estratégias diagnósticas e administrativas de controle da tuberculose em populações institucionalizadas (hospitais, prisões, albergues).**

**Projeto:**

**Viabilidade e Análise econômica da incorporação do teste MGIT960 para o diagnóstico e controle de infecção por tuberculose em pacientes atendidos em Hospitais no Sistema Único de Saúde**

PROCESSO 559081/2009-0

**Proponente: Afrânio Kritski**

**Programa Acadêmico de Tuberculose**

**Faculdade de Medicina da UFRJ**

Novembro 2009

SUMÁRIO

| Item | Página |
| --- | --- |
| Tuberculose no mundo | 1 |
| Incorporação Tecnológica Diagnóstica em TB no mundo | 2 |
| Testes fenotípicos | 2 |
| Detecção de TB resistente | 3 |
| TB no Brasil | 3 |
| Incorporação Tecnológica Diagnõstica no Brasil | 4 |
| Hipótese do estudo | 5 |
| Objetivo Geral | 6 |
| Desfechos | 6 |
| Pacientes e Métodos | 7 |
| Modelos de estudo | 7 |
| Amostra | 7 |
| Seleção (critérios de inclusão e exclusão) | 8 |
| Método – Ensaio Pragmático | 9 |
| Intervenção | 9 |
| Descrição dos testes | 9 |
| Identificação da espécie de M. tuberculosis | 9 |
| Randomização | 10 |
| Procedimentos | 11 |
| Definição de termos | 12 |
| Considerações estatísticas (estudo amostral) | 12 |
| Método Avaliação Econômica | 13 |
| Custo direto | 13 |
| Custo associado a implantação do MGIT960 | 14 |
| Impacto orçamentário da implantação do MGIT960 | 15 |
| Comitê de Monitoramento | 16 |
| Comite Diretor | 16 |
| Equipe de pesquisadores | 16 |
| Cronograma | 17 |
| Orçamento proposto | 18 |
| Justificativa do orçamento proposto | 19 |
| Resultados Preliminares | 20 |
| Referencias Bibliográficas | 21 |
| Anexo 1 – Termo de Consentimento Livre e Esclarecido | 22 |
| Anexo 2- Formulários | 23 |

**1.TB no mundo**

A tuberculose (TB) tem sido causa de preocupação ao longo da história humana tanto no que diz respeito ao seu potencial de ceifar vidas, quanto ao aspecto de sua transmissão e infectividade. Apesar de ser uma doença curável, o processo de transmissão e propagação da TB não foi interrompido de maneira definitiva, notadamente em países em desenvolvimento.

A transmissão da TB tem sido apontada como um dos principais fatores de perpetuação da doença no planeta, associada a fatores como a desigualdade social, o advento da AIDS, e o envelhecimento da população (WHO Report 2008).

A OMS estima que, globalmente, houve um número incidente de 9,27 milhões de casos em 2007, o que significa um aumento quando se considera 9,24 milhões de casos em 2006, 8,3 milhões em 2000, e 6,6 milhões de casos em 1990 (WHO Report 2009).

. Ainda que o número total de casos incidentes de tuberculose esteja aumentado em termos absolutos como resultado do crescimento populacional, o número de casos per capita está em decréscimo. No entanto, a taxa de declínio ainda é considerada lenta, à razão de menos de 1% de declínio ao ano (WHO Report 2009).

Em termos de prevalência, havia cerca de 13,7 milhões de casos de TB em 2007 (206 por 100 mil habitantes), com cerca de 1,3 milhão de mortes entre HIV negativos, e 456.000 mortes adicionais entre HIV positivos.

Com o objetivo de conter o aumento global da TB, foi proposta a estratégia DOTS pela OMS desde 1993, que se baseia no diagnóstico da TB pulmonar por meio da baciloscopia do escarro e o tratamento com esquema encurtado contendo rifampicina (RIF), isoniazida (INH), pirazinamida (PZA) e etambutol (EMB). Entretanto, mesmo com a adoção da estratégia DOTS, a carga de TB tem-se mantido, principalmente nas regiões ou em Unidades de Saúde onde é elevada a prevalência de TB/HIV e TB-MDR.

Um dos fatores primordiais para o surgimento de novos casos é o tempo durante o qual

o paciente portador de TB pulmonar mantém a cadeia de transmissibilidade (Dye e cols 2002). O rendimento diagnóstico da baciloscopia é baixo, principalmente em pacientes com baixa carga bacilar nas amostras respiratórias, em pacientes infectados por HIV ou portadores de outras doenças imunossupressoras.

Estudos com modelos matemáticos mostram que 75% dos casos de TB-MDR necessitam ser detectados e 80% deles precisam apresentar resultado favorável para que diminua a incidência de novos casos de MDR TB, nestas regiões.

No Plano Global STOP TB / OMS de 2006, o aumento do diagnóstico dos casos de TB/HIV e TB-MDR passou, portanto, a ser essencial para o sucesso do programa, nestes locais.

O diagnóstico rápido da TB é importante para se diminuir o tempo de transmissão da doença com conseqüente diminuição do número de pessoas infectadas pelo indivíduo doente.

Apesar da baciloscopia do escarro possuir baixa sensibilidade (40% a 60%), permanece como um dos exames mais utilizados para o diagnóstico de TB, em países em desenvolvimento. (WHO Report 2009). Entretanto, na maioria destes países, na prática, a cultura para micobactérias, cuja sensibilidade é maior (80% a 85%) que a baciloscopia no diagnóstico de TB pulmonar, é realizada em meio sólido com Lowenwstein-Jensen (LJ), e está indicada apenas em casos clínicos selecionados (casos de falência ao tratamento, pacientes com baciloscopia negativa persistente, formas extra-pulmonares) (Ministério da Saúde 2000). O maior problema da cultura em LJ é o longo tempo de incubação (4 a 6 semanas), sendo que o teste de sensibilidade é realizado a partir da cultura e não do espécime clínico, o que requer várias semanas adicionais para a obtenção dos resultados. Na prática, nestes países, com o uso universal apenas da baciloscopia de escarro, cerca de 30% a 40% dos pacientes atendidos nos Serviços de Saúde em países em desenvolvimento, são tratados para TB sem confirmação bacteriológica (Kritski e cols 1997).

**A incorporação de novas tecnologias diagnósticas em TB**

A avaliação de novas tecnologias tem sido também priorizada pela Organização Mundial de Saúde após o lançamento do Plano Global de TB da OMS/STOP em 2006 ([**http://www.who.int/tb/strategy/stop_tb_strategy/en/**](http://www.who.int/tb/strategy/stop_tb_strategy/en/)**).** *Em outubro de 2007, a OMS recomenda o uso do meio líquido para diagnóstico da TB, e da TB resistente* [*http://www.who.int/tb/dots/laboratory/en/index.html*](http://www.who.int/tb/dots/laboratory/en/index.html) *.* Em junho de 2008, a OMS recomenda o uso de testes moleculares para o diagnóstico de TB resistente por meio do parecer do grupo de *experts* da OMS e da UNICEF/UNDP/World Bank/TDR

[http://www.who.int/tb/features_archive/mdrtb_rapid_tests/en/index.html\](http://www.who.int/tb/features_archive/mdrtb_rapid_tests/en/index.html/)

A OMS não recomenda o uso na prática clínica de testes fenotípicos e moleculares *in house*, desenvolvidos em laboratórios de pesquisa, de caráter manual, apesar de apresentarem bons resultados de acurácia (sensibilidade e especificidade) nos locais em que foram desenvolvidos. Pois tais testes não foram validados em outros cenários clínico-epidemiológicos e laboratoriais, e usualmente apresentam baixa reprodutibilidade. Os kits comerciais disponibilizados pelas indústrias demonstram maior reprodutibilidade de seus resultados em diferentes laboratórios. s

**Por outro lado, a OMS enfatiza que, antes da incorporação de tais tecnologias comercializadas na rotina clínica, é necessário que estudos de custo-efetividade e custo-benefício devem ser realizados e avaliados o seu impacto no sistema de saúde em que será utilizado.**

**Testes Fenotípicos**

**Detecção de TB**

Na década de 80, em países industrializados, foi incorporado o teste radiométrico semi-automatizado BACTEC 460 system (Becton Dickinson Diagnostic Systems, Sparks, MD), que utilizava meio líquido e resultou em maior rapidez no diagnóstico da TB. Em razão das limitações do uso de testes radiométricos, por meio de meios líquidos foram desenvolvidos testes não radiométricos manuais e automatizados, como o MGIT (*Mycobacteria Growth Indicator Tube*), baseados na detecção de consumo do O2, e ESP Culture System II que permitem a obtenção de resultado em torno de 10 dias. O MGIT960 já foi incorporado nos laboratórios de referência em países industrializados, considerado como padrão ouro. **Apesar de sua elevada acurácia, ainda não foi adotado em países em desenvolvimento, em razão de requerer insumos e equipamentos mais caros e da ausência de estudos que avaliaram a viabilidade e impacto econômico de sua implantação em condições de rotina nos Programas de Controle de TB.**

Entre os métodos fenotípicos não automatizados baseados em meio líquido a base de ágar, mais apropriados para uso em países em desenvolvimento, destaca-se a técnica *Microscopic Observation Broth Drug Susceptibity Assay* (MODS). Baseia-se num método que, após 8 dias, podem ser observadas no microscópio com lente invertida e com filtro para campo escuro, a visualização do fator corda formado pela micobactéria em crescimento. Até o momento, não foi descrito na literatura revisão sistemática ou meta-análise sobre o uso da técnica MODS para o diagnóstico de TB e de TB resistente. O uso da técnica MODS proporcionou valores preditivos positivos e negativos de 100% e 78,7% em pacientes HIV positivos e de 100% e 82,% em pacientes HIV negativos. O tempo do diagnóstico foi inferior para MODS (6 dias; interquartil de 5 a 7), em comparação com MGIT960 (8,5 dias; interquartil de 6 a 10) e LJ (24 dias; interquartil 18 a 35 dias). Os estudos sobre a técnica MODS sugerem que ela pode ser útil no diagnóstico da TB pois além de apresentar sensibilidade e especifidade similares aos meios de cultura padronizados, o diagnóstico é bem mais rápido. Entretanto, em razão de ser teste in house, requer técnicos de laboratório com elevado grau de proficiência e de biosegurança em razão do uso de meio líquido em placas de Petri.

**Detecção de TB resistente**

Desde 1981, observou-se boa concordância com o método Bactec 460, para a detecção de resistência aos fármacos de primeira linha em relação aos métodos convencionais tanto LJ como Middlebrook 7H10. Houve uma redução importante no tempo de detecção da resistência aos fármacos anti-TB (7 a 14 dias). No final da década de 90, em vários estudos observou-se uma boa concordância na determinação da suscetibilidade de *M.tb* aos fármacos, entre o novo método totalmente automatizado Bactec MGIT960 e o padrão-ouro adotado, apresentando tempo médio de detecção de 7 dias. O órgão regulatório dos Estados Unidos já o aprovou também para a pirazinamida. Novamente, como citado acima, o MGIT960, a**pesar de elevada acurácia no diagnóstico de TB resistente, não foi adotado em países em desenvolvimento, em razão de requerer insumos e equipamentos mais caros e da ausência de estudos que avaliaram a viabilidade e impacto econômico de sua implantação em condições de rotina nos Programas de Controle de TB.**

O Método MB/BacT também tem demonstrado boa concordância para as cepas sensíveis e resistentes a rifampicina e a isoniazida, e apenas para as cepas sensíveis à estreptomicina quando comparados ao método de proporções. O método ESP II é um bom método para a detecção da resistência a rifampicina e a isoniazida; porém não em relação ao etambutol e à estreptomicina. O método ETEST (AB BIODISK, Solna, Suécia) é um teste de sensibilidade quantitativo cujo resultado se obtém 5 a 10 dias após o crescimento de *M.tb* no meio de cultura. ETEST apresenta uma elevada concordância para a detecção de cepas multirresistentes ao ser comparado com o Método de proporções. Por ser um método de baixo custo, requer validação em países em desenvolvimento para o diagnóstico rápido da resistência micobacteriana.

Entre os testes colorimétricos que se caracterizam por leitura visual e que podem ser praticados em laboratórios com recursos técnicos limitados; destacam-se os seguintes: de Indicador de Oxi-Redução do Alamar Blue (MABA); o Método 3-(4,5 dimetiltiazol 2-yl) 2,5-difenil Brometo de Tetrazolio (MTT); e sua variante, o Método Tetrazolium Microplate Assay (TEMA) e o de Redução da Nitratase (NRA). Vários estudos sobre o MABA e o NRA foram publicados demonstrando boa concordância com o Método das Proporções não somente para RMP e INH como também para o EMB e SM. Já o MTT e o TEMA são utilizados para o diagnóstico de detecção de resistência parcial (somente RMP e INH) e tem sido apontado como desvantagem para o MTT, a necessidade de condições adequadas de biossegurança nos laboratórios. Os testes colorimétricos são classificados como métodos in house, e são escassos os estudos que tenham confirmado elevada reprodutibilidade em diferentes laboratórios de países em desenvolvimento. Em razão disto, tais testes não devem ser recomendados para uso de rotina nos laboratórios públicos.

**TB no Brasil**

Brasil ocupa o 18º. lugar entre os 22 países de maior carga de TB em nível mundial. No relatório da OMS sobre as ações de controle de TB em 2009, foi estimado a existência no Brasil de 92.000 casos novos de TB anualmente, com 13.000 pacientes infectados por HIV (14% do total de casos incidentes) e 8.400 mortes por tuberculose sendo que destes, 2.400 pacientes infectados por HIV 28% do total de mortes).

No Brasil, a TB ocupa o quarto lugar como causa de morte por doenças infecciosas e, é a principal causa de mortalidade entre as pessoas infectadas por HIV, mesmo com a adoção universal do esquema HAART (terapia anti-retroviral de elevada eficácia), sem encargos aos pacientes HIV positivos, desde 1997. Apesar da generalização da estratégia DOTS (o pacote básico que está subjacente à Estratégia Stop TB) desde 1998, a incidência da tuberculose tem-se mantido em algumas regiões do país, e 30% dos casos de TB são diagnosticados nos hospitais, onde o diagnóstico é tardio, é infreqüente o acesso a cultura para micobactéria, não há monitoramento efetivo das atividades de controle de TB, são escassas as interações com os programas municipais de controle de TB, e elevada mortalidade e transmissão do bacilo da TB para pacientes e profissionais de saúde.

Entre os 83.089 casos notificados da tuberculose em 2007, existiam 59.166 casos novos de tuberculose pulmonar com mais de 15 anos notificados ao Sistema de Notificação (SINAN). Destes, 19.814 (35,2%) foram notificados sem confirmação bacteriológica, seja porque apresentaram resultados negativos a baciloscopia de escarro ou porque não realizaram tal exame. **Entre os 11.813 casos, com baciloscopia negativa, a cultura preconizada pelo Ministério da Saúde, não foi realizada em 8.073 deles (68,3%).** Entre 9.048 pacientes com alto risco para a tuberculose fármaco-resistente (falência do esquema, recidiva, abandono) os testes de cultura e teste de sensibilidade as drogas preconizados pelo MS, não foram realizadas em 6.268 (69,2%) pacientes. **No que diz respeito à co-infecção TB / HIV, em 2007, entre os 7.334 casos notificados com TB infectados por HIV, cultura para M.tuberculosis também preconizado pelo MS, não foi realizada em 4.541 deles (62%**)(MS-2004). A taxa de mortalidade entre os pacientes com TB infectados por HIV 3,4 vezes maior (15,9%; 1166/7334) do que mortalidade geral (4,6%) observada em casos de TB notificados no SINAN, principalmente em áreas metropolitanas, **em Unidades Hospitalares**, associadas ao retardo diagnóstico. Em 2007, na cidade do Rio de Janeiro, 4.669 adultos com TB, na forma pulmonar, foram notificados ao SINAN, destes, 2.493 (53,4%) apresentaram resultados negativos à baciloscopia, e em 1.101 (23,6%) a baciloscopia não foi realizada. Entre os 2493 casos, com baciloscopia negativa, cultura para M tuberculose não foi realizada em 1.997 delas (80,2%).

A maior taxa de incidência de TB droga sensível (TB-DS), de TB droga resistente (TB-DR) e de TB associada ao HIV (TB-HIV) ocorre nas regiões metropolitanas de Vitória, do Rio de Janeiro e de Porto Alegre, respectivamente. Na análise de dados preliminares descritos na literatura nacional, a taxa de TB multirresistente (TB-MDR) primária varia de 1 a 1,4% nas Unidades Primárias de Saúde (UPS) e de 3,3% a 12% nas Unidades Hospitalares, sendo maior entre pacientes HIV positivos. A maior taxa de letalidade de TB ocorre devido ao diagnóstico incorreto realizado em Unidades Hospitalares, Unidades de Emergência, usualmente sem atividades de controle de TB. Nestes hospitais, apenas 30% dos laboratórios de bacteriologia realizam exames de cultura para micobactéria e raramente disponibilizam resultado do teste de sensibilidade do bacilo da TB às drogas. Estima-se que, em 20% a 30% destes casos, o diagnóstico de TB não foi correto, e a probabilidade de tratamento inadequado ainda é maior em regiões ou em Unidades de Saúde com maior atendimento de pacientes infectados pelo HIV ou portadores de co-morbidades, como em Unidades Hospitalares (Kritski e cols 1997).

**Incorporação Tecnológica em TB no Brasil**

**O uso de novas tecnologias moleculares (kits de PCR) ou fenotípicas (MGIT960 ou MbBact) foram comercializadas no Brasil nos ultimos anos. Tem sido disponibilizadas praticamente na rede privada de laboratórios. Eventualmente foram incluidos em Unidades de Saúde do SUS, mas sem continuidade, em razao da não incorporação na tabela do SIASUS.**

Desde 2002, no Brasil, tem sido proposto por pesquisadores da Rede Brasileira de Pesquisa em TB (REDE-TB) que antes da liberação do registro, sua alocação no mercado nacional e principalmente para sua incorporação pelo Sistema SUS, os novos métodos diagnósticos relacionados a enfermidades de impacto de saúde publica, deveriam ser submetidos a uma avaliação de sua performance e relação de custo efetividade em condições de rotina, através de ensaios clínicos pragmáticos.

**Recentemente, com a *portaria* Nº 2.587, de 30 de outubro de 2008 do Ministério da Saúde (**[**http://bvsms.saude.gov.br/bvs/saudelegis/gm/2008/prt2587_30_10_2008.html**](http://bvsms.saude.gov.br/bvs/saudelegis/gm/2008/prt2587_30_10_2008.html )

**a incorporação de novas tecnologias no Sistema Único de Saúde (SUS) e na ANS devem seguir as recomendações da *Comissão de Incorporação de Tecnologias (CITEC) do Ministério da Saúde vinculada à Secretaria de Ciência, Tecnologia e Insumos Estratégicos, do Ministério da Saúde*).** Cabe a CITEC recomendar a incorporação ou retirada de produtos de saúde com base no impacto da tecnologia no sistema público de saúde e na relevância tecnológica estabelecida com base nos resultados obtidos por estudos de avaliação de tecnologias de saúde, tais como pareceres técnico-científicos, revisões sistemáticas, meta-análise, estudos econômicos e ensaios clínicos pragmáticos.

Usualmente, na avaliação de novos testes diagnósticos, em estudos de acurácia ou performance / demonstração/ viabilidade diagnóstica, os resultados de um teste sob avaliação são comparados com os resultados obtidos por meio de um teste de referência, ambos realizados em um mesmo indivíduo suspeito de possuir a condição de interesse. Novas abordagens sao necessárias, que possibilitem avaliar o impacto clínico e econômico do uso da nova tecnologia em condições de rotina

Ensaios clínicos pragmáticos avaliam intervenções de forma mais próxima possível às condições reais em que elas são aplicadas na prática clínica, servindo de amparo à tomada de decisões em oposição aos ensaios clínicos ditos explanatórios, realizados em condições mais controladas, respondendo a questões sobre se e como uma intervenção funciona, necessárias para obtenção de registro e comercialização de produtos. Estudos pragmáticos, por serem desenvolvidos em condições de rotina, não complicam a prática usual, não competem com as atividades de rotina, e por isso, são baratos e conseguem a colaboração voluntária da equipe

Recentemente, um estudo de validação inter-laboratorial do Bactec MGIT 960 foi finalizado em Laboratórios de referência (Instituto Adolfo Lutz- Lacen SP) e Laboratorios Colaboradores do Sistema Nacional de Laboratórios Públicos do Ministerio da Saúde (Laboratório da UFRJ no Rio de Janeiro e da UFES no Espirito Santo), com resultados satisfatórios (Giampaglia e cols 2007). Observou-se elevada concordância entre a *performance* do MGIT960 e os três métodos considerados padrão ouro para o diagnóstico de TB resistente: a) Método de Proporções, b) Bactec 460, e c) Razão da resistência. Na última década, o MGIT 960 foi validado e aprovado pela ANVISA para os seguintes fármacos: estreptomicina, isoniazida, rifampicina e etambutol.

A partir dos dados acima, **torna-se urgente a realização de estudos que avaliem o impacto de novas tecnologias diagnósticas para TB, TB resistente, e que respondam as demandas estabelecidas pela CITEC de modo a incluir tais tecnologias no SIASUS, propiciando as populações mais desfavorecidas economicamente que procuram os hospitais públicos tenham acesso a novas tecnologias.**

Tais tecnologias devem propiciar o diagnóstico mais precoce de TB e TB resistente em pacientes atendidos em locais fechados (hospitais, prisões, albergues) de maneira a favorecer a redução do tempo de exposição dos companheiros de enfermaria/de cela e da comunidade a pacientes infectados, propiciar controle de internação em ambiente apropriado o mais rápido possível, com os impactos previsíveis de tais medidas na morbidade e mortalidade da TB, assim como na cadeia de transmissibilidade da doença.

**Hipótese do estudo**

Teste fenotípico totalmente automatizado com modelo integrado de transformação do MGIT960 é robusto o suficiente para ser utilizado em laboratórios de referencia e locais (hospitais) e permite aumentar significativamente as taxas de detecção de casos de tuberculose, bem como para encurtar tempo para o início do tratamento, em comparação com o algoritmo diagnóstico convencional. Manuseio de paciente suspeito de TB baseado no teste MGIT960, e liberação do resultado enquanto o paciente ainda encontra-se internado na Unidade de Saúde, diminui a morbi-mortalidade dos pacientes com TB, bem como a transmissão da TB em nível intra-hospitalar (possibilita adoção de medidas administrativas no controle de infecção), e os custos e as barreiras para o diagnóstico e tratamento anti-TB.

**Locais do estudo**

O projeto será realizado no laboratório de Micobacteriologia do Hospital Universitário Clementino Fraga Filho da UFRJ

**Objetivo - Geral :** Avaliar o impacto clínico e econômico do uso da técnica MGIT960 na rotina de diagnostica de pacientes suspeitos de Tuberculose atendidos em hospital universitário e Unidade de nível secundário na sudeste do Brasil.

**Desfechos:**

The primary outcome was defined as the proportion of patients in each group whose initial prescription changed by the second month after randomization. The ‘change’ could be initiating or stopping TB treatment.

**1. Desempenho clínico (fase de viabilidade)**

• comparar a proporção de pacientes que mudam o tratamento decorridos dois meses depois da inclusão no estudo no grupo de intervenção e no grupo da rotina
• Estimar sensibilidade e especificidade nas amostras com baciloscopia negativa em relação aos resultados obtidos com a cultura

**2. Desempenho operacional (fase de viabilidade e custo efetividade)**

• Avaliar robustez dos reagentes e equipamentos do teste MGIT960 (temperatura, poeira, irregularidades energia, taxas de contaminação, indeterminado resultados)
• Identificar necessidades na manutenção e suporte ao cliente, bem como formação mínima na área de recursos humanos
• Avaliar a confiança dos médicos no uso dos resultados fornecidos pelo teste MGIT960

**3. Impacto (fase de viabilidade e avaliação econômica)**

- Avaliar o tempo para a detecção da tuberculose e para iniciar o tratamento anti-TB
- Avaliar o número de dias e a proporção de pacientes com baciloscopia negativa e cultura positiva que ficaram fora do isolamento respiratório, durante a internação, até o resultado do teste.
- Avaliar os resultados do tratamento anti-TB (proporção de tratamento completos, mortalidade, abandono, reações adversas)
- Elaborar um modelo de custo-efetividade para as alternativas de diagnóstico da tuberculose pulmonar, a partir dos casos detectados, casos encaminhados para tratamento e casos negativados;
- Estimar o custo unitário direto do teste MGIT960 e do Lowenstein Jensen;
- Estimar o custo da implantação do teste MGIT 960 no SUS;
- Estimar o custo médio associado a detecção e monitoramento do paciente com TB pulmonar ou extrapulmonar internado no hospital
- Estimar o custo por atividade associado a detecção e monitoramento do paciente com TB pulmonar ou extrapulmonar internado no hospital
- Estimar o custo médio associado à detecção e ao monitoramento do caso de tuberculose pulmonar;
- Utilizar os resultados de efetividade do teste MGIT960 e da Lowenstein Jensen fornecidos pelo ensaio clínico pragmático;
- Estimar a razão de custo-efetividade incremental do teste MGIT960 quando comparado à cultura pelo Lowenstein Jensen, expressa em casos detectados, casos encaminhados para tratamento e casos negativados;

Estimar o impacto orçamentário da incorporação do teste MGIT960 para o SUS.

- Estimar o impacto orçamentário da incorporação do teste MGIT960 para o Hospital Universitário Clementino Fraga Filho da UFRJ e no HUES.

**3. Pacientes e Métodos**

## Modelo de estudo de viabilidade e impacto nas ações de controle de infecção (performance em condições de rotina)

Será realizado um ensaio clínico pragmático da técnica MGIT-960 no diagnóstico de tuberculose em pacientes internados consecutivamente, com suspeita de TB em hospital da UFRJ e Unidade de nível Secundária do Rio de Janeiro (Policlínica Augusto Amaral Peixoto).

**Modelo de avaliação econômica**

Será realizada uma análise de custo-efetividade com o objetivo comparar os custos e a efetividade das duas estratégias diagnósticas para o SUS, utilizando um modelo de decisão analítico por meio da construção de árvore de decisão, com probabilidades de eventos considerando os horizontes temporais do estudo.

**Modelo do impacto orçamentário**

Será elaborado um modelo de análise de decisão do impacto no orçamento do teste molecular teste MGIT 960 e que terá como premissas: i) os indicadores epidemiológicos da doença; e ii) o custo da incorporação do teste no SUS, considerando as diferenças na organização da rede de serviços de saúde.

## Amostra de estudo

A população de referência será constituída de todos os indivíduos que sejam internados no HUCFF/UFRJ e atendidos na Policlínia Augusto Amaral Peixotofig 1). A população de estudo será constituída por aqueles que sejam considerados elegíveis para o estudo ou seja, todos os pacientes internados no HUCFF/UFRJ e atendidos no ambulatório da PAAP-SMS que estejam sob suspeita de serem portadores de TB.

Aqueles que atenderem aos critérios de inclusão no estudo constituirão a amostra do estudo (Figura 1). Em 2008, no HUCFF-UFRJ, foram notificados 165 casos de TB, sendo 42 (25%) infectados por HIV, 106 (64%) na forma pulmonar.. Entre os pacientes com TB pulmonar, 36 (34%) Baar +, 35 (33%) Baar - / cult+ e 55 com diagnostico de probabilidade. Em 2008, na PAAP-SMS , foram notificados 308 casos de TB, sendo 15 (5%) infectados por HIV, 280 (90%) na forma pulmonar. Entre os pacientes com TB pulmonar, 240 com baciloscopia positiva, 44 com baciloscopia negativa e cultura positiva e 24 com diagnostico de probabilidade.

## Seleção de amostra

### *Critérios de inclusão*

Serão incluídos neste estudo todos os indivíduos cujos dados clínicos e laboratoriais sejam sugestivos de tratar-se de primeiro episódio ou recidiva de TB ativa (caso suspeito):

- que aceitem participar do estudo e assinem o Termo de Consentimento livre e esclarecido, quer por livre expressão da vontade, quer representado legalmente.
- que tenham idade maior que 18 anos.

### *Critérios de exclusão*

Serão excluídos do estudo os indivíduos que:

- sejam portadores de TB ativa em tratamento antes da aleatorização;
- sejam portadores de resultados de cultura que apontem para o isolamento de *M. tuberculosis* nos seis meses anteriores à aleatorização.

Figura 1: Esquema de seleção de amostra para o estudo

**População de referência**

(Pacientes atendidosno HUCFF/UFRJ e na PAAP

**População de estudo (elegíveis)**

(Dados clínicos e laboratoriais sugestivos de primeiro episódio ou

recidiva de TB ativa)

**Critérios de exclusão**

Indivíduos que:

- Sejam portadores de TB ativa antes da aleatorização
- Possuam cultura positiva para M. tuberculosis nos seis meses anteriores à aleatorização

**Critérios de inclusão**

**AMOSTRA DE ESTUDO**

Indivíduos que:

- Aceitem participar do estudo
- Idade maior que 18 anos

## Método Ensaio Clínico Pragmático

### *Intervenção*

No HUCFF/UFRJ e na PAAP-SMS, após randomização, espécimes clínicos de pacientes internados serão submetidos ao MGIT-960 ou cultura em meio sólido de Lowestein Jensen (LJ) (padrão ouro).

### *Descrição dos testes*

Espécimes respiratórios e não respiratórios, naturalmente contaminados, serão coletados em pote descartável. Os espécimes deverão ser entregues ao laboratório até o próximo dia útil e serão submetidos à baciloscopia após coloração de Ziehl-Neelsen. Após descontaminação, serão cultivados pelo método BACTEC MGIT 960 ou em LJ. Culturas contaminadas por outros microrganismos serão designadas como contaminadas e consideradas como negativas na análise dos dados. Nos espécimes clínicos positivos à cultura dos pacientes incluídos será realizado o teste de sensibilidade do *M. tuberculosis* para INH, RIF, SM e EMB pelos testes padrão ouro ou pelo BACTEC MGIT 960. As culturas positivas serão avaliadas pelo teste indireto das proporções (uma cepa/paciente).

### Identificação do M. tuberculosis –

### Métodos convencionais (LJ) - As cepas selecionadas serão identificadas como *M. tuberculosis,* com base nas características das colônias (rugosas, cremes e opacas), na capacidade de produzir niacina e nitrato redutase e termo inativação da catalase.

**Identificação do complexo *M. tuberculosis* pelo Sistema BACTEC MGIT 960** – No fundo de cada tubo há um sensor de fluorescência. O nível de fluorescência que cada tubo emite corresponde à quantidade de oxigênio consumido pelo organismo, que é proporcional ao número de bactérias presentes no tubo. Quando o oxigênio é consumido ocorre uma reação de oxi-redução fazendo com que a fluorescência aumente. Essa fluorescência pode ser detectada através de um transiluminador com lâmpada UV no comprimento de onda de 365 nm. Quinhentos microlitros de sedimento tratado dos espécimes clínicos serão semeados em cada um dos tubos de MGIT sem e com ácido para-nitro benzóico (PNB) na concentração de 500μg/mL. O resultado da identificação do complexo *M. tuberculosis* será definido com base na inibição de seu crescimento nos meios de cultura MGIT contendo PNB e crescimento (positivação) no tubo de MGIT controle. Será preparada uma suspensão padronizada das cepas de *M. tuberculosis*, *M. bovis*, *M. africanum* e de outras espécies de micobactérias crescidas em meio sólido. Quinhentos microlitros das suspensões serão semeados em meio MGIT 960 com 500μg/mL de PNB e meio controle sem PNB. Os frascos serão homogeneizados, identificados por meio da leitura do código de barras, e introduzidos em estante específica do instrumento BACTEC MGIT 960. O resultado da identificação do complexo *M. tuberculosis* será definido com base na inibição de seu crescimento nos meios de cultura MGIT contendo PNB e crescimento (positivação) no tubo de MGIT controle. O resultado é dado num prazo médio de 15 dias, dependendo do crescimento bacteriano.

**Teste de Sensibilidade a drogas** **pelo Sistema BACTEC MGIT 960** – As cepas de *M. tuberculosis* serão subcultivadas em tubo MGIT até indicação de resultado positivo no Sistema BACTEC MGIT 960. Cinco tubos de MGIT (7 mL) serão identificados e dispostos na estante (AST Set Carrier), na seguinte sequência: GC (controle - Sem drogas), STR (Estreptomicina), INH (Isoniazida), RIF (Rifampicina) e EMB (Etambutol). A seguir um volume correspondente a 0,8 mL do BACTEC MGIT SIRE Supplement será adicionado a cada tubo. Assepticamente, utilizando uma micropipeta, serão pipetados 100 μl das soluções-estoque de cada droga nos 4 tubos, de maneira a atingir as concentrações finais apresentadas na tabela 1. A partir das culturas de *M. tuberculosis* com crescimento nos tubos de MGIT será então preparada uma diluição 1:100, pela adição de 0,1 mL das culturas em 10 mL de solução salina estéril. Após a homogeneização das suspensões bacterianas, será inoculado 0,5 mL da suspensão diluída (1:100) em um tubo de meio de cultura MGIT (controle sem drogas). Nos demais tubos, contendo as drogas (INH, RMP, EMB e SM) será inoculado 0,5 mL da suspensão não diluída. Os frascos serão homogeneizados, identificados por meio da leitura do código de barras, e introduzidos na estante do teste de sensibilidade no instrumento BACTEC MGIT960. Os resultados (sensibilidade ou resistência às drogas) serão definidos de acordo com os critérios estabelecidos pelo fabricante (Becton Dickinson) com base no algoritmo do programa de computador do Sistema.

**Teste de sensibilidade às drogas pelo método das proporções –** Será realizado com base no método indireto descrito por Canetti, Rist e Grosset (1963). A partir do crescimento bacteriano em meio sólido, sem drogas, será feita uma suspensão, utilizando o maior número de colônias possível, em tubo 16 x 150mm contendo cerca de 10 pérolas de vidro e aproximadamente 0,5 mL de água destilada estéril. O tubo será agitado em vórtex por 20 a 30 segundos para homogeneizar a suspensão. A seguir serão gotejadas lentamente à suspensão água destilada estéril até obter a turvação correspondente ao tubo número 1 na escala de Mac Farland ou de uma suspensão padrão de BCG de 1mg/mL. A partir desta suspensão padronizada serão efetuadas diluições decimais até 10^-6^ e semeados 0,1mL nos tubos controles sem drogas e nos tubos com drogas. Após a semeadura, os tubos serão colocados inclinados horizontalmente em uma bandeja de tal forma que o inóculo se distribua sobre toda a superfície do meio, mantendo-os com a tampa frouxa por 24-48 horas para secar o inóculo. Os tubos serão então incubados na posição vertical à 37ºC por 28 dias. Após este período a leitura do teste e interpretação dos resultados serão realizados de acordo com as recomendações de Normas Bacteriológicas do Manual Técnico do Ministério da Saúde.

No Laboratório de Micobacteriologia do HUCFF- UFRJ e do NDI-UFES, as diversas tarefas serão distribuídas entre os técnicos, Assim, um técnico é responsável pelo processamento diário dos espécimes clínicos pelo método de Kubica e semeadura do sedimento em meio de LJ, outro técnico é responsável pela realização dos testes de sensibilidade às drogas pelo método das proporções, um terceiro pela semeadura e teste de sensibilidade nos tubos do método BACTEC MGIT 960, dos espécimes deste e de outros estudos, etc. Deste modo não há possibilidade do técnico ser induzido a favorecer a leitura positiva de um ou de outro método. Soma-se a esta divisão de tarefas, o fato da leitura do MGIT960 ser totalmente automatizada, sendo seu resultado impresso pelo computador do aparelho em fita com impressão térmica, que será xerocada e arquivada em lugar próprio.

### *Randomização – Cegamento*

Este é um ensaio aberto. O profissional de saúde que avalia de rotina os pacientes sob suspeita de TB ativa, convidado a participar de modo voluntário do estudo, uma vez que decida pela inclusão do paciente no estudo, solicitará um envelope que contém na sua parte externa, quatro perguntas, a serem respondidas por um médico:

a) na sua opinião qual a probabilidade do paciente apresentar TB (alta/intermediária ou baixa);

b) a escolha acima foi baseada nos resultados de exames radiológicos;

c) na sua opinião este paciente deve iniciar tratamento anti-TB agora (sim ou não)

d) na sua opinião qual a probabilidade deste paciente ter TB resistente (alta, intermediária ou baixa).

Apenas após preenchidas as quatro perguntas, o profissional de saúde abrirá o envelope e, no seu interior, identificará qual o método diagnóstico que deverá ser solicitado para o paciente. Os envelopes serão numerados em ordem crescente e a indicação dos testes diagnósticos no seu interior terá sido previamente randomizada por profissional não participante do estudo. Este procedimento procura garantir o ocultamento da randomização. As perguntas, respondidas antes da abertura do envelope, permitirão checar o sucesso da aleatorização.

### *Procedimentos*

Na rotina de investigação de pacientes sob suspeita de TB serão coletadas em formulário padronizado as seguintes informações:

a) dados sócio-demográficos;

b) revisão dos órgãos e sistemas do corpo humano;

c) exposição à TB, doença e história de tratamento;

d) história patológica pregressa;

e) exame físico padronizado;

f) resultado de radiografias de tórax;

g) resultado do teste de HIV;

Espécimes respiratórios incluem escarro espontâneo, escarro induzido e lavado broncoalveolar. Espécimes não respiratórios incluem material de biópsia, e líquidos (pleurais, sinoviais, cefaloraquidiano, etc). Não serão coletados espécimes clínicos fora da rotina de investigação diagnóstica para TB no hospital. Seguindo a rotina, as amostras clínicas serão encaminhadas aos laboratórios de micobacteriologia no próximo dia útil. Em caso de amostras clinicas de pacientes que foram aleatorizados para o MGIT, o laboratório deverá ser informado que apenas o MGIT deve ser realizado.

### *Operacionalização do estudo (viabilidade)*

**Coleta de dados** - Todas as informações disponíveis nos prontuários e fichas de rotina serão coletadas e repassadas para um formulário específico. Os nomes dos pacientes serão coletados apenas uma vez e esta informação será guardada separadamente em formulário que não contenha dados clínicos ou laboratoriais. Um número para identificação do paciente será usado em todos os formulários que contenham resultados. Serão utilizadas as informações coletadas na rotina do Programa de Controle de TB Hospitalar, segundo o Manual de Normas divulgado em 1998, pois eles são avaliados clinicamente e monitorados continuamente durante as primeiras 8 semanas da fase de investigação diagnóstica e os que recebem o diagnóstico de TB ativa são monitorados mensalmente até o final do tratamento medicamentoso. Informações coletadas na rotina de acompanhamento do Programa de Tuberculose Hospitalar são obtidas nas semanas 2, 4, e 8 e serão registradas nos formulários próprios deste estudo.

Os testes de laboratório necessários para o diagnóstico de TB serão pedidos no formulário de requisição do teste de laboratório que será impresso em papel sensível à pressão de folhas múltiplas. No laboratório, os resultados do teste serão gravados no formulário da requisição e as folhas serão separadas e distribuídas como segue: a folha inferior permanece no laboratório, a folha do meio é retornada à clínica e a folha superior vai à sala de computador para a introdução de dados. Na rotina do PCTH, tem-se realizado uma avaliação da qualidade da coleta dos dados clínicos duas vezes por ano, em 10% dos pacientes incluídos nos 6 meses precedentes. Deste modo, os resultados primários e os computadorizados destes pacientes serão revisados. O acesso aos arquivos de dados e dos registros do estudo será limitado ao pessoal do estudo, incluindo a equipe de campo e visitadores da Universidade Federal do Rio de Janeiro.

**Definição de termos**

**Ensaio clínico pragmático** – ensaio clínico que visa avaliar as intervenções na forma o mais próximo possível das condições reais em são aplicadas na prática clínica (condições de rotina). Um ensaio clínico pragmático aleatorizado objetiva avaliar o uso de novos métodos diagnósticos na rotina dos serviços de saúde.

**Caso suspeito de TB** – todo paciente sob suspeita de TB que apresente sinais/sintomas e alterações laboratoriais, histopatológicas ou radiográficas compatíveis

**Caso confirmado de TB –** paciente que possua ao menos um resultado de cultura positivo para *M. tuberculosis* em LJ ou MGIT60.

**Caso provável de TB** – paciente que apresente resultados negativos à cultura com as seguintes características:

a – resposta clínica ao tratamento (definido como melhor à radiografia de tórax, diminuição da tosse e/ou ganho de peso de 3% em relação ao início do tratamento) e

b – ausência de diagnóstico alternativo

**Pacientes “não TB**” – pacientes que não tenham sido classificados com caso confirmado ou provável de TB

**Considerações estatísticas**

**Cálculo amostral**

O cálculo do tamanho da amostra para o desfecho primário de mensuração da proporção de pacientes que tiveram seu tratamento modificado após receber o resultado de cultura para o MTb baseou-se em uma coleta de dados realizada pela equipe do estudo para o estudo piloto (ver abaixo), que revelou que cerca de 9,0% dos pacientes iniciaram o tratamento anti-TB após o resultado da cultura em MGIT960 e 3,7% com LJ (padrão ouro). Para detectar uma diferença entre os testes de pelo menos 5,3%, com erro alfa de 5% e poder de 80%, será necessário recrutar 736 pacientes. **Assumindo uma perda de 10% - a amostra final será de 800** **suspeitos de TB** incluídos nos dois hospitais. Já foram incluídos 220 suspeitos no HUCFF. Pretende-se incluir os 580 suspeitos restantes nos dois hospitais, nos próximos 18 meses.

**Análise dos dados de viabilidade / performance**

A análise será baseada nos grupos conforme a alocação aleatória, isto é, por intenção de tratamento e será feito inicialmente como proporções e comparada usando o teste do qui-quadrato ou o teste exato de Fisher, quando apropriado. Se as características basais forem significativamente diferentes entre os grupos que utilizaram o BD960 e de controle, o ajuste pela regressão logística multivariada será realizado. A eficácia do BACTEC MGIT960 na detecção de *M. tuberculosis* em espécimes respiratórios de pacientes com suspeita de TB será avaliada usando a definição clínica de TB. O tempo de isolamento do Mtb pelas duas técnicas será avaliado pelo modelo de Kaplan-Meier e a diferença entre os testes será avaliada pelo teste de log rank. Para os desfechos contínuos serão calculadas a média, mediana e desvio padrão e avaliadas pelo teste t de Student para distribuição normal.

**Projeto Avaliação econômica**

**Estratégias diagnósticas que serão avaliadas**

Será avaliado o teste MGIT960 para a detecção de *M. tuberculosis* (MTB) em comparação com a estratégia convencional, cultura para micobactéria com meio sólido Lowenstein Jensen. Este teste também é capaz de detectar a resistência aos medicamentos anti-TB de primeira linha (rifampicina, isoniazida, etambutol e estreptomicina) contudo neste estudo esta condição não será avaliada.

**Perspectiva da análise**

A perspectiva da análise do estudo é a do SUS, órgão responsável pelo pagamento dos serviços de saúde e pelo financiamento do diagnóstico e tratamento da tuberculose.

**Desfechos de interesse**

A análise será realizada considerando os seguintes desfechos de interesse: caso detectado, caso encaminhado para tratamento e caso negativado.

**Horizonte temporal**

Serão considerados os seguintes horizontes temporais: o primeiro compreende o período da entrada do paciente no sistema de saúde até o momento do diagnóstico; o segundo compreende o momento em que o paciente é internado para investigação diagnóstica até o isolamento respiratório para evitar a transmissão de TB intra-hospitalar, considerando a sua entrada no sistema; e o terceiro relaciona-se com o período de monitoramento do caso de tuberculose pulmonar em até 6 meses, a partir do encaminhamento para tratamento, e da transmissão entre os contatos institucionais.

**Análise de custos**

Esta etapa do estudo irá estimar os custos diretos não médicos e médicos do diagnóstico da tuberculose pulmonar e do período de monitoramento.

**Custo direto do diagnóstico da tuberculose pulmonar considerando as duas estratégias**

**a) Custo direto unitário do teste MGIT960**

O método do micro-custo (*micro-costing*) será aplicado, pois permite que se realize um inventário detalhado dos recursos consumidos e sua quantificação (recursos e custo unitário).

Nesta etapa serão considerados os seguintes itens de custos: compra do equipamento (com valores anualizados), material de consumo, recursos humanos (salários) e capacitação de pessoal.

O valor do salário dos recursos humanos terá como referência a Tabela de Remuneração dos Servidores Públicos. A decisão de utilizar esta referência salarial se deve ao fato desta tabela homogeneizar as diferenças regionais. Para o cálculo será considerada a média salarial das classes. Caso os profissionais não sejam dedicados completamente à realização do diagnóstico, será considerado o tempo gasto para a realização de cada teste para o cálculo dos salários.

Em relação ao custo do treinamento de recursos humanos para o teste MGIT 960, conforme o protocolo do ensaio clínico o tempo necessário é de sete a dez dias para os laboratórios de referência. Para outros locais onde o teste pode ser realizado, esta informação não está disponível e deverá ser buscada. Para estimar o custo do treinamento serão levantados: tempo gasto, necessidade de treinamento por período (se anual, bianual ou outro), passagens, diárias e outro tipo de remuneração exigido pela equipe responsável pelo treinamento, passagens e diárias para os alunos, dentre outros itens necessários para a realização dessa atividade.

As informações para o cálculo desses custos serão obtidas da Becton and Dickinson e outras fontes que se façam necessárias para a estimativa do custo.

**Custo direto unitário da cultura com Lowenstein Jensen**

Para estimar o custo direto da cultura para miocobacteria com Lowenstein Jensen também será aplicado o método do micro-custeio, considerando os insumos e recursos necessários para a realização do diagnóstico.

**Custo associado à implantação do teste MGIT 960 no SUS**

Para a introdução do teste MGIT960 no SUS, serão estimados os custos referentes aos requisitos necessários para sua implantação e funcionamento. Serão estimados os custos relativos à:

- Estrutura física: espaço dedicado ao teste, ambiente climatizado, instalações elétricas e segurança do equipamento (roubo);

- Equipamentos: compra e manutenção preventiva e corretiva, suporte ao cliente, incluindo assistência técnica e estoque de peças no Brasil;

- Insumos e reagentes: quantidade necessária para atender a previsão de testes a serem realizados anualmente fornecidos e não fornecidos pela Becton and Dickinson

- Material de expediente e equipamentos de apoio;

- Recursos humanos e capacitação; e

- Outros recursos necessários para a introdução da tecnologia nos serviços de saúde.

As informações serão obtidas através da Becton and Dickinson e outras fontes necessárias.

**Custo médio e por atividade (CBA)da detecção e do monitoramento do caso de tuberculose pulmonar (custos médicos diretos)**

O custo médio da detecção e o monitoramento do caso até o 6º mês será estimado conforme o que é preconizado pelo Manual de Normas para o Controle da Tuberculose do Ministério da Saúde. Serão considerados os seguintes itens de custos: consultas, exames e medicamentos. Os dados serão obtidos através de bases nacionais (Tabelas do SUS, SIH-SUS, SIA-SUS, Banco de Preços do Ministério da Saúde) e outras que se fizerem necessárias. Serão apropriados os mesmos itens pelo método CBA.

Serão considerados os custos associados à falência do tratamento, às complicações associadas à tuberculose e ao re-tratamento. Para os pacientes HIV positivos não será estimado o custo de complicações associadas à doença principal.

**Razão de custo-efetividade**

A comparação entre os custos e a efetividade das duas estratégias diagnósticas será dada pela razão de custo-efetividade incremental.

**Taxa de desconto**

A taxa de desconto utilizada será de 5% conforme as recomendações das Diretrizes Metodológicas para Estudos de Avaliação Econômica de Tecnologias para o Ministério da Saúde.

**Análise de sensibilidade**

Na realização de análises econômicas a imprecisão de determinados parâmetros do modelo e a ausência de dados requerem que o pesquisador defina alguns pressupostos para reduzir o tamanho da incerteza gerada por essas limitações. Neste estudo para avaliar o impacto das variações dos parâmetros que podem repercutir nos resultados, a análise de sensibilidade considerará: taxa de desconto, medidas de acurácia e de custos.

**Impacto orçamentário do teste MGIT 960**

Esta etapa do projeto pretende mensurar o impacto orçamentário da introdução do teste MGIT 960 para o diagnóstico da tuberculose em nível hospitalar. Serão considerados os custos em relação aos seguintes aspectos: adequação do equipamento à rede de serviços de saúde, compra, manutenção preventiva e corretiva, suporte técnico (equipe especializada, tempo de atendimento e estoque de peças), insumos (reagentes e outros) necessários para a realização do teste e recursos humanos. Também serão incluídos parâmetros epidemiológicos e associados aos serviços de saúde a partir das especificidades de sua organização e distribuição no SUS.

**Cenários a serem definidos**

Os cenários serão criados a partir de análise de sensibilidade e o pressuposto é de que a nova tecnologia poderá substituir a estratégia convencional sob uma perspectiva mais conservadora ou mais ampla.

**Perspectiva**

A perspectiva da análise do impacto orçamentário será a do SUS, responsável pelas estratégias de diagnóstico e tratamento da tuberculose pulmonar e pelo financiamento da introdução da nova tecnologia.

**Horizonte temporal**

O horizonte temporal definido será de um ano devido ao processo orçamentário do SUS ser anual.

**Custos**

Os custos associados à incorporação da tecnologia terão como referência o item *3.1.8. Análise de Custos* e serão estimados para o período de um ano. Também será identificado se a introdução da tecnologia altera o uso de recursos e a capacidade do sistema de saúde para disponibilizá-la.

# Comitê de monitoramento de dados

Embora este estudo não represente um risco potencial a seus participantes, uma vez que o recrutamento pode se estender por um prazo prolongado, um comitê de monitoramento de dados, independente dos investigadores e financiadores do estudo, será constituído com a finalidade de avaliar a necessidade de acesso aos dados antes de seu término. As reuniões deste comitê são convocadas por seu presidente, quando houver qualquer condição que possa por em risco a segurança dos participantes, ou se houver razões para a interrupção precoce do estudo. Sempre que solicitado, o comitê diretor dará acesso aos dados ao CMD. O comitê poderá recomendar a interrupção do estudo, mas tal decisão deverá ser tomada pelo comitê diretor. Os membros deste comitê são:

Cláudio J Struchiner (estatístico –PROCC-FioCruz) - presidente

Luiz AB Camacho (epidemiologista -ENSP-FioCruz

Jose Roberto Lapa e Silva (pneumologista – HUCFF-UFRJ)

# Comitê diretor do estudo

O papel do comitê diretor é supervisionar o estudo, assegurar que ele está sendo conduzido de acordo com as principais regulamentações e que não há desvios importantes do protocolo. É deste comitê a responsabilidade por qualquer decisão em relação ao estudo. Os membros deste comitê são:

Dr Afrânio Kritski – Tisio-Pneumologista - UFRJ; Dra Gisele Huf – epidemiologista – INCQS – FioCruz; Dra Leila Souza Fonseca – Microbiologista - UFRJ – Presidente do Comitê Diretor; Dra Anete Trajman – Universidade Gama Filho; Dra Valeria Rolla – infectologista – IPEC-Fiocruz; Sr Ézio Tavora – Vice-Coordenador da Area de Mobilização Social da Rede TB

**Equipe do Estudo**

**Coordenação da Avaliação da viabilidade e performance do estudo em condições de rotina – Uso de ensaio clinico pragmático**: Programa Acadêmico de Tuberculose PAT- Faculdade de Medicina /FM da Universidade Federal do Rio de Janeiro/UFRJ

- Afrânio Kritski – prof da Faculdade de Medicina da UFRJ – coordenador do PAT

- Adriana da Silva Resende Moreira – enfermeira, coordenadora do estudo – PAT

- Alberto Santa Cruz Coimbra – pesquisador – mestrando – PAT

- Anna Grazia Marsico – chefe do laboratorio de Micobacteria do HUCFF

- Gisele Huf – pesquisadora da Fiocruz – expert em Ensaios Clínicos Pragmáticos

- Leila de Souza Fonseca – prof titular – Coordenadora da Area Diagnostica do PAT

-- Maria Armanda Vieira – doutora em Pneumologia – pesquisador do PAT

- Paulo Albuquerque da Costa – doutor em Pneumologia – pesquisador do PAT

**Coordenação e Supervisão Avaliação Econômica**:

- Claudia Vater – médica e economista - Instituto de Educação Saúde Coletiva da UFRJ

**Avaliação pelo CEP**

Este projeto foi aprovado pelo Conselho de Ética em Pesquisa (CEP) do Hospital Universitário Clementino Fraga Filho, da Universidade Federal do Rio de Janeiro, como parte integrante do projeto “Ensaio clínico pragmático e avaliação de custo-efetividade de técnicas para o diagnóstico de Tuberculose Sensível e Resistente em pacientes atendidos em dois hospitais na região sudeste do Brasil”, sob o número 020/07.

**Cronograma do estudo**

|  | Out-Dez 2008 | Jan-  Fev 2009 | Marco  set 2009 | Out a dez 2009 | Jan a maio 2010 | Junho a dez 2010 | Jan a maio 2011 | Junho a out 2011 | Nov a dez 2011 |
| --- | --- | --- | --- | --- | --- | --- | --- | --- | --- |
| Revisão bibliográfica + | Xxx |  |  |  |  |  |  |  |  |
| elaboração do projeto e questionários | xxx | Xxx |  |  |  |  |  |  |  |
| Coleta de dados (estudo piloto) |  |  | Xx | Xx |  |  |  |  |  |
| Coleta dados complementares |  |  |  |  | xx | xx | xx | xx |  |
| Analise de dados – viabilidade |  |  |  | Xx |  |  |  | xx | Xx |
| Visita a laboratórios e serviços de diagnóstico |  |  |  | Xxxx |  |  |  |  |  |
| Estimativa dos parâmetros do modelo |  |  |  |  | xxxx |  |  |  |  |
| Cálculo da medida de efetividade |  |  |  |  | xxxx |  |  |  |  |
| Avaliação de custo-efetividade |  |  |  |  | xxxx | Xxxx | Xxxx | xxxx |  |
| Análise do impacto orçamentário |  |  |  |  | xxxx | Xxxx | Xxxx | xxxx | xxxx |
|  |  |  |  |  |  |  |  |  |  |

# Financiamento

Edital Doenças Negligenciadas Faperj . Estratégias para aumentar a detecção de TB resistente em Unidades de Saúde na região metropolitana do Rio de Janeiro. Processo E-26/111.608/2008.

**Orçamento proposto**

| Item | Especificação | Quantidade | Período | Valor unitário (R$) | Valor total (R$) |
| --- | --- | --- | --- | --- | --- |
| 1) Serviços de Terceiros – Pessoa Física:  a) Coordenação e supervisão do projeto (ensaio pragmático) | - Enfermeiro (n=2, um em cada hospital)- Economista | 2 | 18 meses | 1.800,00 | 64.800,00 |
| 2) Serviços de Terceiros – Pessoa Física:  a) Coordenação e supervisão do projeto (avaliação econômica) | Economista (n=2) | 2 | 9 meses | 2.500,00 | 45.000,00 |
| b) Coordenador Geral do Estudo | - Pesquisador clínico | 1 | 24 meses | 2.0000,00 | 48.000,00 |
| c) Serviço de digitação | - digitará todos os dados coletados em 800 pacientes | 1 | 9 meses | 20,00/quest. | 16.000,00 |
| 2) Serviços de Terceiros – Pessoa Jurídica: | - Serviços de fotocópia  - Compra de artigos científicos |  |  | 4.000,00 | 4.000,00 |
| 3) Passagens | Rio-Vitoria-Rio | 6 |  | 1.000,00 | 6.000,00 |
| 4) Diárias (Tabela Servidor Público Federal) | Vitoria ou Rio | 20 |  | 224,20 | 4.484,00 |
| 5) Material permanente  a) Software Tree Age Pro Health Care® CUSTEIO | Licença do Software Tree Age Pro Health Care® | 2 | - | 2.821,50 | 5.643,00* |
| 5) Material Permanente  b) Freezer | Freezer – 20c | 2 | - | 1.500,00 | 3.000,00 |
| 5) Material Permanente  c) centrifuga | Centrifuga refrigerada | 2 | - | 15.000,00 | 30.000,00 |
| 5) material permanente  d) computadores | Compaq - | 2 | - | 1.500,00 | 3.000,00 |
| 6) Material de consumo:  Suprimentos | Toner, pendrive, papel para impressão | - | - |  | 4.000,00 |
| **7) Material de consumo: laboratorio**** | Vidriarias, meios de cultura, |  |  |  | **7.000,00** |
| **Sub-total** | - | - | - | - | **233.927,00** |
| **Bolsistas** | DTI | 2 | 24 meses |  |  |
| **Total geral** |  |  |  |  |  |

* Preço do software Tree Age Pro Health Care®: US$ 1,485.00 (<http://www.treeage.com/products/healthPricing.html>).

*Cotação do dólar: US$ = R$ 1,90.

** - custos para MGIT960 serão cobertos pela empresa Becton and Dickinson

Justificativa do orçamento proposto

Para a realização do projeto será necessária a participação de profissionais com experiência na área de laboratório de micobacteriologia, de ensaios clínicos pragmáticos, avaliação de tecnologias em saúde, análise econômica e modelagem estatística. Estes profissionais irão coordenar e supervisionar a execução do projeto com o apoio de bolsistas, cujo requisito para a participação será ter experiência nas áreas de pesquisa clínica e operacional, engenharia clínica, laboratório de análise clínica e avaliação econômica em saúde. A equipe irá realizar levantamento de dados na literatura, coleta de dados no serviço, análise e elaboração de relatórios. A equipe será formada também com o objetivo de se estruturar um grupo neste campo do conhecimento, contribuindo para criar uma *expertise* em ensaios clínicos pragmáticos e avaliação econômica de tecnologias em saúde na área de diagnóstico e tratamento da tuberculose no Brasil. Ademais, pretende contribuir para orientar a tomada de decisão sobre a alocação de recursos e a formulação de políticas em um cenário de crescimento do controle da doença do país.

Os serviços de digitação, suprimentos e fotocópia serão necessários para operacionalizar a realização do projeto. A compra de artigos científicos ou de relatórios de agência de avaliação não disponibilizados pelos Periódicos Capes poderá ser necessária, já que há etapas da análise econômica que serão baseadas em dados da literatura.

O software *Tree Age Pro Health Care*® e compuadores serão necessários para o repasse dos dados nos dois sites e para a construção dos modelos, realizar a análise dos resultados da avaliação de performance, custo-efetividade e análise de sensibilidade. O orçamento propõe a compra de duas licenças perpétuas e cada uma ficará instalada em um computador do NATS/IFF e em um computador do PEB/COPPE.

A aquisição de 2 freezer a -20C e centrifuga refrigerada será necessário para manter a qualidade na execução dos procedimentos das amostras clínicas nos laboratórios de referencia de micobacteriologia dos 2 hospitais universitários.

A compra de material de consumo faz-se necessária devido ao gasto para a impressão dos relatórios no NATS/IFF e na COPPE/UFRJ. Estas duas instituições darão como contrapartida o tempo de trabalho e salário de seus profissionais (PEB/COPPE) e o uso de suas instalações físicas e computadores (PEB/COPPE e Unidade de Pesquisa Clínica/NATS/IFF).

# Dados preliminares

Na fase piloto, durante o período de fevereiro a setembro de 2009, a equipe do estudo piloto triou 279 pacientes internados no HUCFF/UFRJ, e incluiu no estudo 220 pacientes, distribuídos entre os braços, na quantidade de 112 pacientes para o braço MGIT960, e 108 pacientes para o braço L-J.

Suspeitos de TB: BAAR+ = 8/220 (3,7%)

Cultura+ = 21 /220 (9,5%)

Proporção de HIV+ = 57/220 (25,9%)
 Imunossupressão =  29/220 (13,2%)

O resultado da cultura em ambos os braços, é mostrado na Tabela 1:

Tabela 1:

**Resultado da Cultura:**  279 pacientes triados - 220 pacientes incluídos

(MGIT960: 112; LJ: 108)

| Braços | 0 – 10 Dias | 11 -20 Dias | 21 – 30 Dias | 31 – 40 Dias | 41 – 50 Dias | 51 – 60 Dias |
| --- | --- | --- | --- | --- | --- | --- |
| MGIT  112 | Pos: 10 | Pos: 3 | Pos:1 | - | Neg: 94 | - |
| LJ  108 | - | Pos:1 | Pos:3 | Pos:3 | Pos:2 | Neg:95 |

Diagnóstico precoce de TB (igual ou inferior a 10 dias após a internação) foi observado em 8,9% (10/112) dos pacientes submetidos ao teste MGIT960 e nenhum no grupo que fez uso de LJ. Diagnóstico inferior a 20 dias foi observado em 11,6% (13/112) no grupo submetido ao MGIT960 e em 0,9% (1/108) no grupo submetido ao LJ.

A Tabela 2 mostra os dados relativos ao desfecho primário deste estudo:

Tabela 2: **Mudança de Conduta:**  279 pacientes triados - 220 pacientes incluídos (MGIT960: 112; LJ: 108)

| Braços | 0 – 10 Dias | 11 -20 Dias | 21 – 30 Dias | 31 – 40 Dias | 41 – 50 Dias | 51 – 60 Dias |
| --- | --- | --- | --- | --- | --- | --- |
| MGIT  10/112  (8,9%) | 6 | 3 | 1 | - | - | - |
| L-J  4/108  (3,7%) | - | - | 1 | 2 | 1 | - |

A mudança de conduta após o resultado da cultura pelo teste MGIT960 foi de 9,0%, enquanto que pelo teste LJ foi de 3,7%.

Tais números preliminares apontam na direção de diagnóstico mais precoce e maior proporção de mudança de tratamento quando usado o método MGIT960.

Espera-se, portanto, corroborar os dados apresentados como preliminares após coleta da amostra do estudo, de maneira a responder à questão da aplicabilidade do teste MGIT960 na prática rotineira. A simplicidade propiciada pelos ensaios clínicos pragmáticos permite a sua adaptação situações clínicas, sem alterar a prática rotineira.

**Referências bibliográficas**

- Brasil. Ministério da Saúde. Secretaria de Ciência, Tecnologia e Insumos Estratégicos. Departamento de Ciência e Tecnologia. Diretrizes Metodológicas: estudos de avaliação econômica de tecnologias em saúde. Brasília, 2009.
- Corbett E L, Watt C J, Walker N, Maher D, Williams B G, Raviglione M C, Dye C. The growing burden of tuberculosis: global trends and interactions with the HIV epidemic. Arch Intern Med. 2003 May 12;163(9):1009-21.
- Drummond MF et al. Methods for the economic evaluation of health care programmes. Oxford: Oxford University Press, 1997
- Dye C, Garnett G P, Sleeman K, Williams B G. Prospects for worldwide tuberculosis control under the WHO DOTS strategy. Directly observed short-course therapy. Lancet 1998;352:1886-91.
- Dye C, Williams B G, Espinal M A, Raviglione M C. Erasing the world's slow stain: strategies to beat multidrug-resistant tuberculosis. Science. 2002 Mar 15;295(5562):2042-6.
- Kritski A L, de Jesus L S R, Andrade M K et al. Retreatment tuberculosis cases: factors associated with drug resistance and adverse outcomes. Chest 1997;111:1162-7.
- Giampaglia CMS, Martins MC, Vieira GBO, Vinhas SA, da Silva Telles MA, Palaci M, Marsico AG, Hadad DJ, Mello FCQ, Kritski A, Siddiqi S, Fonseca LS. Multi-center Evaluation of Automated Bactec MGIT 960 System for testing susceptibility of *M. tuberculosis* as compared with BACTEC 460TB, Proportion and Resistance Ratio Methods in Southeast of Brazil**.** [Int J Tuberc Lung Dis.](javascript:AL_get(this, 'jour', 'Int J Tuberc Lung Dis.');) 2007 Sep;11(9):986-91.
- Mauskopf J. et al. Principles of Good Practice for Budget Impact Analysis: Report of the ISPOR Task Force on Good Research Practices – Budget Impact Analysis. Value in Health 2007; 10 (5).
- Moreira A S R, Castro C B A, Castro P A, Vieira M A M, Mello F C Q, Aguiar F S, Marsico A S , Vieira G B O, Ruffino-Netto A, Fonseca L S, Huf G, Kritski A L. 2009. A Pragmatic Clinical Trial To Evaluate Performance of Diagnostic Tests in Tuberculosis: MGIT960 vs Lowenstein Jensen. Preliminary Results. Am J Respir Crit Care Med 179:A3196
- Normas técnicas para o Programa de Controle da Tuberculose. 5th ed. Rev. e ampliada. Brasilia: Ministério da Saúde, 2000.
- Pfyffer, G. E., C. Cieslak, H. M. Welscher, P. Kissling, and S. Rüsch-Gerdes. 1997. Rapid detection of mycobacteria in clinical specimens by using the automated BACTEC 9000 MB system and comparison with radiometric and solid-culture systems. J. Clin. Microbiol. 35:2229–2234.
- Pfyffer, G. E., H. M. Welscher, P. Kissling, C. Cieslak, M. J. Casal, J. Gutierrez, and S. Rüsch-Gerdes. 1997. Comparison of the Mycobacteria Growth Indicator Tube (MGIT) with radiometric and solid culture for recovery of acid-fast bacilli. J. Clin. Microbiol. 35:364–368.
- Siddiqi, S. H. 1989. BACTEC TB system. Product and procedure manual, revision B. Becton Dickinson Diagnostic Instruments System, Towson, Md.
- [SBPT] Sociedade Brasileira de Pneumologia. 2009. III Consenso Brasileiro de Tuberculose. Bras Pneumol. 2009;35(10):1018-1048.
- Somoskövi, and P. Magyar. 1999. Comparison of the Mycobacteria Growth Indicator Tube with MB Redox, Löwenstein-Jensen, and Middlebrook 7H11 media for recovery of mycobacteria in clinical specimens. J. Clin. Microbiol. 37:1366–1369.
- Tenover FC, Crawford JT, Huebner RE, Geiter LJ, Horsburgh CR, Good RC. The Resurgence of tuberculosis: is your laboratory ready? J Clin Microbiol 1993;31:767-70.
- Tortoli, E., P. Cichero, M. G. Chirillo, M. R. Gismondo, L. Bono, G. Gesu, M. T. Simonetti, G. Volpe, G. Nardi, and P. Marone. 1998. Multicenter comparison of ESP Culture System II with BACTEC 460TB and with Lowenstein-Jensen medium for recovery of mycobacteria from different clinical specimens, including blood. J. Clin. Microbiol. 36:1378–1381.
- Trueman P. et al. Developing Guidance for Budget Impact Analysis. Pharmacoeconomics 2001; 19 (6): 609-621.
- [WHO] World Health Organization. 2009. Global Tuberculosis Control. WHO Report. Geneva, Switzerland
- Zanetti, S., F. Ardito, L. Sechi, M. Sanguinetti, P. Molicotti, G. Delogu, M. P. Pinna, A. Nacci, and G. Fadda. 1997. Evaluation of a nonradiometric system (BACTEC 9000 MB) for detection of mycobacteria in human clinical samples. J. Clin. Microbiol. 35:2072–2075.
- Zwarenstein M, Treweek S, Gagnier JJ, Altman DG, Tunis S, Haynes B, Oxman, AD, Moher D, for the CONSORT and Pragmatic Trials in Healthcare (Practihc) groups.. Improving the reporting of pragmatic trials: an extension of the CONSORT statement . BMJ; 337: 1-8, 2008

Anexo 2. TERMO DE CONSENTIMENTO LIVRE E ESCLARECIDO

**Ensaio clínico pragmático da técnica MGIT-960 no diagnóstico de Tuberculose em pacientes atendidos em um Hospital Universitário e numa Policlínicde Janeiro**

No Hospital Universitário Clementino Fraga Filho da UFRJ e na Policlínica Augusto Amaral Peixoto (Guadalupe) está sendo realizado um estudo em indivíduos sob suspeita de tuberculose em atividade para avaliar qual o melhor teste diagnóstico a ser utilizado. O diagnóstico rápido da tuberculose é fundamental para diminuir complicações para o paciente e também para diminuir o tempo de transmissão da doença para os familiares.

Novos testes diagnósticos já tiveram sua eficácia comprovada através de estudos, entre eles, o teste MGIT 960. Embora a um custo mais elevado, ele possibilita um diagnóstico mais rápido que o teste rotineiramente utilizado, o teste de Lowenstein-Jensen. Durante o estudo, o material para exame (escarro ou outro), será submetido a um dos dois testes (MGIT ou L-J). A escolha do teste é feita de forma aleatória, como num sorteio, e o estudo irá apontar o melhor teste a ser utilizado na rotina dos hospitais, em termos de custo-efetividade e benefícios para o paciente.

Todos os desfechos avaliados pelo estudo já constam da rotina de avaliação dos pacientes no hospital e, por isso, a participação no estudo não envolve qualquer procedimento ou exame adicional para o paciente. Dessa forma, riscos ou desconfortos que possam ocorrer não diferem entre os que participam ou não do estudo. A participação ou não no estudo também não traz qualquer implicação para o tratamento do paciente, todos recebem o melhor cuidado disponível no hospital. O estudo será coordenado pelos pesquisadores Dr. Afrânio Kritski e Dra. Leila Souza Fonseca, que podem ser contatados pelos telefones: (21) 2550-6903 ou (21) 2562-2426.

A PARTICIPAÇÃO NA PESQUISA É VOLUNTÁRIA e toda informação pessoal é mantida em sigilo.

________________________ ______________________ _________________

Assinatura do voluntário Nome completo N^o^ do prontuário

(ou responsável legal)

___________________________ ______________________ ____/_____/_____

Assinatura do entrevistador Nome do entrevistador Data

Rapid
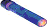
 TB

# *Ensaio Clínico*

**Formulário de Desfechos**

## Formulário de desfechos

## IDENTIFICAÇÃO

## Número da randomização

1. Números de identificação hospitalar:

Prontuário PCTH Amb./PCTH

1. Nome completo
2. Sexo F M 5. Data de nascimento

OBS.:

**RANDOMIZAÇÃO**

1. Data ____/____/______ 7. Hora ____:____ hs.

1. Método MGIT L-J
2. Responsável
3. 1° material colhido: _____________________ 11. Data da coleta ____/____/______

OBS.:

**DESFECHO PRINCIPAL**

1. Resultado da cultura Negativo MTB MNTB

Data: ____/____/______

1. Resultado TSA Sensível Resistente

Data: ____/____/______

1. Mudança de conduta com o paciente? (No período de até 3 meses a partir da entrada do primeiro material)

Sim Não

1. Descreva

## Formulário de desfechos (Cont.)

Rapid
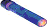
 TB

# *Ensaio Clínico*

**Formulário de Desfechos**

**BACTERIOLOGIA**

| **MR:** |  | |  | **HISTÓRICO BACTERIOLÓGICO** | | | | | | | | | |  | |
| --- | --- | --- | --- | --- | --- | --- | --- | --- | --- | --- | --- | --- | --- | --- | --- |
|  | Material | |  |  | |  |  |  | | |  | |  | | |
|  | Data | | *__/__/____* | *__/__/____* | | *__/__/____* | *__/__/____* | | | | *__/__/____* | | *__/__/____* | | |
|  | **BAAR** | |  |  | |  |  | | | |  | |  | | |
|  | N° Cepa | |  |  | |  |  | | | |  | |  | | |
| **CULTURA** | | Resultado |  |  | |  |  | | | |  | |  | | |
|  |  | Tempo Dias |  |  | |  |  | | | |  | |  | | |
|  |  | Data CBK | *__/__/____* | *__/__/____* | | *__/__/____* | *__/__/____* | | | | *__/__/____* | | *__/__/____* | | |
|  |  | Identificação |  |  | |  |  | | | |  | |  | | |
|  |  | TSA* |  |  | |  |  | | |  | | |  | | |
|  |  | Data TSA | *__/__/____* | *__/__/____* | | *__/__/____* | *__/__/____* | | | | *__/__/____* | | *__/__/____* | | |
|  |  | Qualidade |  |  | |  |  | | |  | | |  | | |
|  |  | Material |  |  | |  |  |  |  |  |  |  |  | | |
| OBS: | | |  | |  |  |  | |  | | |  | | |  |

*SENSÍVEL = escrever “sensível”; RESISTENTE = escrever as siglas de que medicações há resistência: R - Z - S - E - O - Et.

OBS.:

**CO-MORBIDADES**

| **CO-MORBIDADES** | 1 = Doença renal cronica | 6 = Transplante | 11 = Silicose |
| --- | --- | --- | --- |
|  | 2 = Hepatopatia | 7= NA | 12 = DPOC |
|  | 3 = Alcoolismo | 8 = Ign | 13= Doença difusa |
|  | 4 = Uso corticóides | 9= Diabetes mellitus NID | 14=Outra (citar) |
|  | 5 = Neoplasia Maligna | 10 = Diabetes melitus ID |  |

**Formulário de desfechos (Cont.)**

**HIV**

1. Positivo Negativo Não oferecido Não realizado
2. Data do anti-HIV ___/___/______
3. Valor CD4 19. Data CD4: ___/___/______
4. Uso de anti-retroviral: Sim Não
5. Data início anti-retroviral: ___/___/______

OBS.:

**DESFECHOS GERAIS I**

1. Causa da perda do caso
2. Causa do óbito
3. Falência clínica
4. Falência radiológica

Rapid
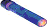
 TB

# *Ensaio Clínico*

**Formulário de Desfechos**

**DESFECHOS GERAIS II**

1. Listar exames realizados não específicos para TB (envolvidos e direcionados para o diagnóstico)

| EXAMES | EFEITOS ADVERSOS | DATAS |
| --- | --- | --- |
|  |  |  |
|  |  |  |
|  |  |  |
|  |  |  |
|  |  |  |
|  |  |  |

1. Efeitos adversos ao tratamento anti-TB* e não-TB*

| N° efeitos adversos  Tratamento | Relação* | Datas |
| --- | --- | --- |
|  |  |  |
|  |  |  |
|  |  |  |
|  |  |  |
|  |  |  |
|  |  |  |
|  |  |  |
|  |  |  |
|  |  |  |
|  |  |  |
|  |  |  |
|  |  |  |
|  |  |  |

| 2. Exantema c/mucosite | 17. Prurido |
| --- | --- |
| 3.Vômitos confusão mental | 18. Anorexia |
| 4.Icterícia | 19. Náuseas |
| 1. 5.Hepatoxicidade | 20. Vômitos |
| 6.Vertigem, nistagmo | 21. Epigastralgia |
| 7.Hipoacusia | 22.Dor articular |
| 8.Crises convulsivas | 23.Neurite periférica |
| 9.Encefalopatia tóxica e coma | 24.Euforia |
| 10.Neurite ótica | 25.Insônia, sonolência |
| 11.Psicose | 26. Ansiedade |
| 12.Trombocitopenia, leucopenia, eosinofilia, anemia hemolítica, agranulocitose e vasculite | 27. Cefaléia |
|  | 28. Acne |
|  | 29.Hiperuricemia c/ ou s/sintomas |
| 13.Reação sistêmica (choque ou púrpura) | 30.Febre |
| 14. Nefrite intersticial | 31.Hipotensão ortostática |
| 15.Rabdomiólise c/mioglobinúria e insuf. renal | 32. Ginecomastia |
| 16.Exantema ou prurido | 33.Outros |

1. Listar exames específicos para TB

| EXAMES | SIM | NÃO | EFEITOS ADVERSOS | DATAS |
| --- | --- | --- | --- | --- |
| 1.Radiografia de torax |  |  |  |  |
| 2.TC |  |  |  |  |
| 3.RNM |  |  |  |  |
| 4.Outro exame radiológico |  |  |  |  |
| 5.Outros (descrever)  *BFC/Toracocentese/Punção lombar/Biópsias/EE/EI | | | | |

DESFECHOS GERAIS III

| 29.Data da internação |  |
| --- | --- |
| 30.Data da alta hospitalar |  |
| 31.Data de início do tratamento para TB |  |
| 32.Data do término do tratamento para TB |  |
| 33.Data do início de medicação não-TB antes da confirmação diagnóstica |  |
| 34. Data do término de medicação não-TB antes da confirmação diagnóstica |  |
| 35.Esquema inicial tto. TB |  |
| 36.Esquema final tto. TB |  |

Rapid
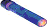
 TB

# *Ensaio Clínico*

**Formulário de Desfechos**

**DIAGNÓSTICO FINAL**

37. É tb? Sim Não

38. Se não é TB, qual é o diagnóstico?

**DESFECHOS PARA TUBERCULOSE**

39. Tipo de diagnóstico Clínico Bacteriológico

40. Sítio da TB: ___________________________________________

41. Peso em grama

_________________ 1° mês / Data: ___/___/______ (1ª consulta PCTH)

_________________ 2° mês / Data: ___/___/______ (consulta 2° mês)

42. Melhora da tosse (TB pulmonar) no 1° e 2° mês?

1° mês Sim Não Data: ___/___/______

2° mês Sim Não Data: ___/___/______

43. Conversão bacteriológica ao tratamento anti-TB até 60 dias?

Sim Não Data: ___/___/______

OBS.:
